# Mechanistically Distinct Mouse Models for *CRX*-Associated Retinopathy

**DOI:** 10.1371/journal.pgen.1004111

**Published:** 2014-02-06

**Authors:** Nicholas M. Tran, Alan Zhang, Xiaodong Zhang, Julie B. Huecker, Anne K. Hennig, Shiming Chen

**Affiliations:** 1 Ph.D. program in Molecular Genetics and Genomics, Washington University in Saint Louis, Saint Louis, Missouri, United States of America; 2 College of Arts & Sciences, Washington University in Saint Louis, Saint Louis, Missouri, United States of America; 3 Department of Ophthalmology and Visual Sciences, Washington University in Saint Louis, Saint Louis, Missouri, United States of America; 4 Department of Developmental Biology, Washington University in Saint Louis, Saint Louis, Missouri, United States of America; Stanford University School of Medicine, United States of America

## Abstract

Cone-rod homeobox (CRX) protein is a “paired-like” homeodomain transcription factor that is essential for regulating rod and cone photoreceptor transcription. Mutations in human *CRX* are associated with the dominant retinopathies Retinitis Pigmentosa (RP), Cone-Rod Dystrophy (CoRD) and Leber Congenital Amaurosis (LCA), with variable severity. Heterozygous *Crx Knock*-*Out* (*KO*) mice (“*+/−*”) have normal vision as adults and fail to model the dominant human disease. To investigate how different mutant CRX proteins produce distinct disease pathologies, we generated two *Crx Knock*-*IN (K-IN)* mouse models: *Crx^E168d2^* (*“E168d2”*) and *Crx^R90W^* (*“R90W”*). *E168d2* mice carry a frameshift mutation in the CRX activation domain, *Glu168del2*, which is associated with severe dominant CoRD or LCA in humans. *R90W* mice carry a substitution mutation in the CRX homeodomain, *Arg90Trp*, which is associated with dominant mild late-onset CoRD and recessive LCA. As seen in human patients, heterozygous *E168d2* (“*E168d2/+*”) but not *R90W* (*“R90W/+”*) mice show severely impaired retinal function, while mice homozygous for either mutation are blind and undergo rapid photoreceptor degeneration. *E168d2/+* mice also display abnormal rod/cone morphology, greater impairment of CRX target gene expression than *R90W/+* or *+/−* mice, and undergo progressive photoreceptor degeneration. Surprisingly, *E168d2/+* mice express more mutant CRX protein than wild-type CRX. *E168d2neo/+*, a subline of *E168d2* with reduced mutant allele expression, displays a much milder retinal phenotype, demonstrating the impact of *Crx* expression level on disease severity. Both CRX^[E168d2]^ and CRX^[R90W]^ proteins fail to activate transcription *in vitro*, but CRX^[E168d2]^ interferes more strongly with the function of wild type (WT) CRX, supporting an antimorphic mechanism. *E168d2* and *R90W* are mechanistically distinct mouse models for *CRX*-associated disease that will allow the elucidation of molecular mechanisms and testing of novel therapeutic approaches for different forms of *CRX-*associated disease.

## Author Summary

The transcription factor Cone-Rod Homeobox (CRX) plays a central role in regulating gene expression of rod and cone photoreceptors, the primary light sensing cells of the retina. Mutations in the human *CRX* gene have been associated with the retinal degeneration diseases Retinitis Pigmentosa (RP), Cone-Rod Dystrophy (CoRD) and Leber Congential Amaurosis (LCA). These diseases cause progressive and permanent loss of vision, vary widely in age of onset and severity, and are currently untreatable. To understand how mutations in *CRX* cause distinct forms of retinal disease, we have genetically engineered mice to carry human disease-causing mutations in their *Crx* gene. These mouse lines accurately recapitulate distinct forms of *CRX*-associated disease, demonstrating that different classes of CRX mutations are responsible for phenotype variability in humans. We have characterized the pathology of these mice and identified critical mechanisms of disease. In addition, we have discovered that modifying the level of mutant protein had a dramatic effect on disease pathology in one mutant model, suggesting that targeted therapy against the mutant CRX could be an effective treatment strategy. These mouse models will allow for the testing of novel therapeutic strategies for retinal diseases caused by *CRX* mutations.

## Introduction

**Figure 1:**
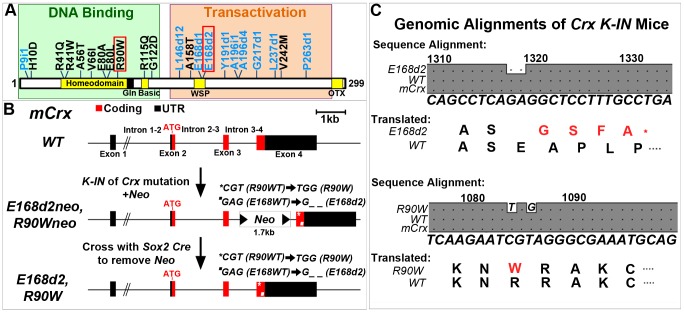
Generation of mechanistically distinct *Knock*-*IN* (*K-IN*) mouse lines for *CRX*-associated disease: *E168d2*, *E168d2neo*, *R90W* and *R90Wneo*. **A.** Diagram of CRX protein showing regions associated with DNA binding (green box) and transactivation (orange box) and mutations associated with human retinopathies. These mutations mainly fall into two classes: frameshift deletions and insertions in the transactivation region (blue text) and amino acid substitutions within the DNA binding region (black text). Two mutations (marked by red box) were selected for generating knock-in mouse models: *E168d2* was predicted to generate a truncated protein that interferes with wild-type CRX function; *R90W* was predicted to generate a protein with reduced ability to bind DNA. **B.** Diagram of mouse *Crx* locus showing gene structure and strategy for generating *Crx E168d2* and *R90W K-IN* lines. *E168d2neo* and *R90Wneo* each carry the indicated targeting construct containing *loxP*-flanked *Neo* cassette in Intron 3–4 as a selection marker. The final *E168d2* and *R90W* lines were generated from the respective ‘*Neo+’* sublines by *Sox2-Cre*-mediated excision in germline. **C.** Germline transmission of *K-IN* constructs was confirmed by Sanger sequencing of genomic DNA from homozygous *E168d2/d2*, *R90W/W* and *WT* mice and aligned to genomic *mCrx*. Shaded ‘**•**’ in the grey boxes indicate fully conserved sequences, unshaded ‘**•**’ denote deletions, and letters indicate base pair substitutions. Gene position (above alignment), consensus sequence (below alignment) and translated amino acid sequence are shown. Amino acid changes in *E168d2* and *R90W* are shown in red text with ‘*’ indicating the novel stop codon in *E168d2*. Further generations of *E168d2* and *R90W* mice were genotyped by allele specific PCR amplification of genomic DNA (Figure S1).

CRX (Accession: AAH53672.1) is an Otd/OTX-like ‘paired’ homeodomain transcription factor that is preferentially expressed in vertebrate rod and cone photoreceptor cells in the retina and pinealocytes in the brain [1], [2]. CRX plays an essential role in the development and maintenance of functional mammalian rod and cone photoreceptors [3]. Previous studies show that CRX acts as a transcriptional activator [1] [4]–[6] by interacting with co-activators, promoting histone acetylation at target gene promoters [7] [8] and mediating enhancer/promoter intrachromosomal looping interactions [9] of target photoreceptor genes. *Crx* encodes a 299 amino acid protein that contains a homeodomain (HD) near its N-terminus that is responsible for DNA binding (Figure 1A) [1] [10]. The HD is followed by glutamine rich (Gln), basic, WSP and OTX-tail motifs. The C terminal region of CRX (from the basic to the OTX-tail domains) is required for transactivation activity [4]. CRX interacts with transcription co-regulators including the rod-specific transcription factors NRL (Accession: NP_006168.1) [11] [12], NR2E3 (Accession: AAH41421.1) [13] [14], and general co-activator proteins GCN5, CBP and p300 (Accessions: AAC50641.1, AAC17736.1, NP_001420.2, respectively) [7] to coordinately control photoreceptor gene expression. In the homozygous *Crx Knock*-*Out* mouse (“−/−”), photoreceptors fail to form outer segments (OS), a highly specialized photoreceptor organelle which contains visual pigment opsins and other proteins required for phototransduction [15] [16]. As a result, −/− photoreceptors do not function [3], form abnormal synapses [17], and undergo progressive degeneration [3]. Gene expression profile studies showed that −/− mice have severely reduced expression of many photoreceptor specific genes [18]–[20]. Most of these genes are direct CRX targets as detected by ChIP-seq analyses of the genomic CRX binding profile in the mouse retina [21].

Mutations in human *CRX* (NCBI Reference Sequence: NG_008605.1) have been associated with autosomal dominant forms of the retinal degenerative diseases Retinitis Pigmentosa (adRP), Cone-Rod Dystrophy (adCoRD) and Leber Congenital Amaurosis (adLCA), with different ages of onset and severity [12] [22]–[45]. *CRX* is the only gene associated with all three diseases [22] [23] [26] [43], demonstrating its central role in rod and cone integrity. However, null mutations in *CRX* may not be responsible for severe dominant disease. A null mutation in *CRX*, *P9ins1*, was associated with LCA in a heterozygous patient but the patient's father, a carrier of *P9ins1*, had a normal ocular phenotype suggesting either recessive or multigenic inheritance [44]. The heterozygous *Knock-Out* mouse (“*+/−*”), also shows only a slight delay in photoreceptor development and fails to model severe forms of dominant human disease [3]. The phenotypes of the human heterozygous null mutation and the +/− mouse phenotype suggest that haploinsufficiency is unlikely to underlie the severe forms of dominant *CRX*-associated disease.

Dominant disease-causing human *CRX* mutations primarily fall into two classes (Figure 1A): frameshift mutations (blue text) mostly in the transactivation domains and amino acid substitution mutations (black text) mostly within the DNA binding homeodomain. Both classes are expected to produce mutant forms of CRX protein that are pathogenic. Truncated CRX proteins resulting from the frameshift mutations *E168d1*, *E168d2*, *A196d4* and *G217d1* lost the ability to transactivate the promoter of *Rhodopsin* (*Rho*) in HEK293 cell transient transfection assays, but are expected to bind DNA normally since CRX 1–107, a complete activation domain truncation mutant, retained CRX target binding activity [4]. It was predicted that these truncated mutant proteins could interfere with the function of WT CRX by an antimorphic mechanism and cause a severe dominant retinal phenotype. Supporting this hypothesis, *E168d1*, *E168d2*, *G217d1* and several other truncation mutations were linked to early onset (0–20 years) severe adCoRD/adLCA [22]–[33] [36]–[40] and *A196d4* was associated with adult onset adCoRD [42] . Furthermore, rescue experiments of the *otd^uvi^* phenotype in *Drosophila* demonstrate the CRX truncation mutation *I138^fs48^* possessed dominant-negative activity on target gene expression [46], providing experimental evidence for an antimorphic mechanism for this class of *CRX* mutations.

Four substitution mutations in the homeodomain: *R41W*, *R41Q*, *R90W* [11] [45] [47], and *K88N* [12], also reduced the ability of CRX to bind to and transactivate the *Rhodopsin* promoter. *R41Q* and *R90W* both reduced CRX:NRL protein interaction [11], while *K88N* additionally interfered with basal NRL-mediated transcription [12]. *R41W*, *R41Q*, and *R90W* were predicted to represent hypomorphic alleles associated with either recessive or less severe dominant forms of disease, while *K88N* was predicted to possess antimorphic activity on NRL function causing a stronger phenotype. Supporting this hypothesis, *R41W*, *R41Q*, *R90W* and several other substitution mutations were associated with late onset (∼40–60 years old) adCoRD [22] [23] [33] [36] [42] [45], while *K88N* was associated with adLCA [12]. A patient homozygous for *R90W* was also diagnosed with autosomal recessive LCA [45]. In contrast, four other substitution mutations *E80A* [22] [23] [33] [39], *A56T* [31], *A158T* and *V242M* [42] did not lose DNA binding or transactivating activity [47] and were associated with early onset adCoRD/LCA. *In vivo* rescue experiments in *Drosophila* also demonstrate that *E80A* but not *R90W* or *K88N* possesses some dominant-negative activity on *Rh5* expression [46]. Collectively these experiments support our hypothesis that substitution mutations may cause disease through several distinct mechanisms.

Currently, there is no treatment strategy for *CRX*-associated diseases. Establishing animal models that accurately recapitulate different disease mechanisms is critical for developing and testing novel therapeutic approaches. Here we report the generation of two mechanistically distinct *Knock-IN (K-IN)* mouse models, each carrying a different class of *CRX* mutation, and present a detailed morphological, functional and biochemical characterization of these mouse models. The frameshift mutation *E168d2* produces a severe dominant phenotype through an antimorphic mechanism, while the substitution mutation *R90W* produces a very mild late-onset ‘CoRD-like’ phenotype in heterozygotes and ‘LCA’-like disease in homozygotes. Furthermore, the expression level of a mutant allele can dramatically affect the disease phenotype, providing insight into potential treatment strategies.

## Results

### Generation of *Crx E168d2* and *R90W K-IN* mutant mouse models

**Figure 2:**
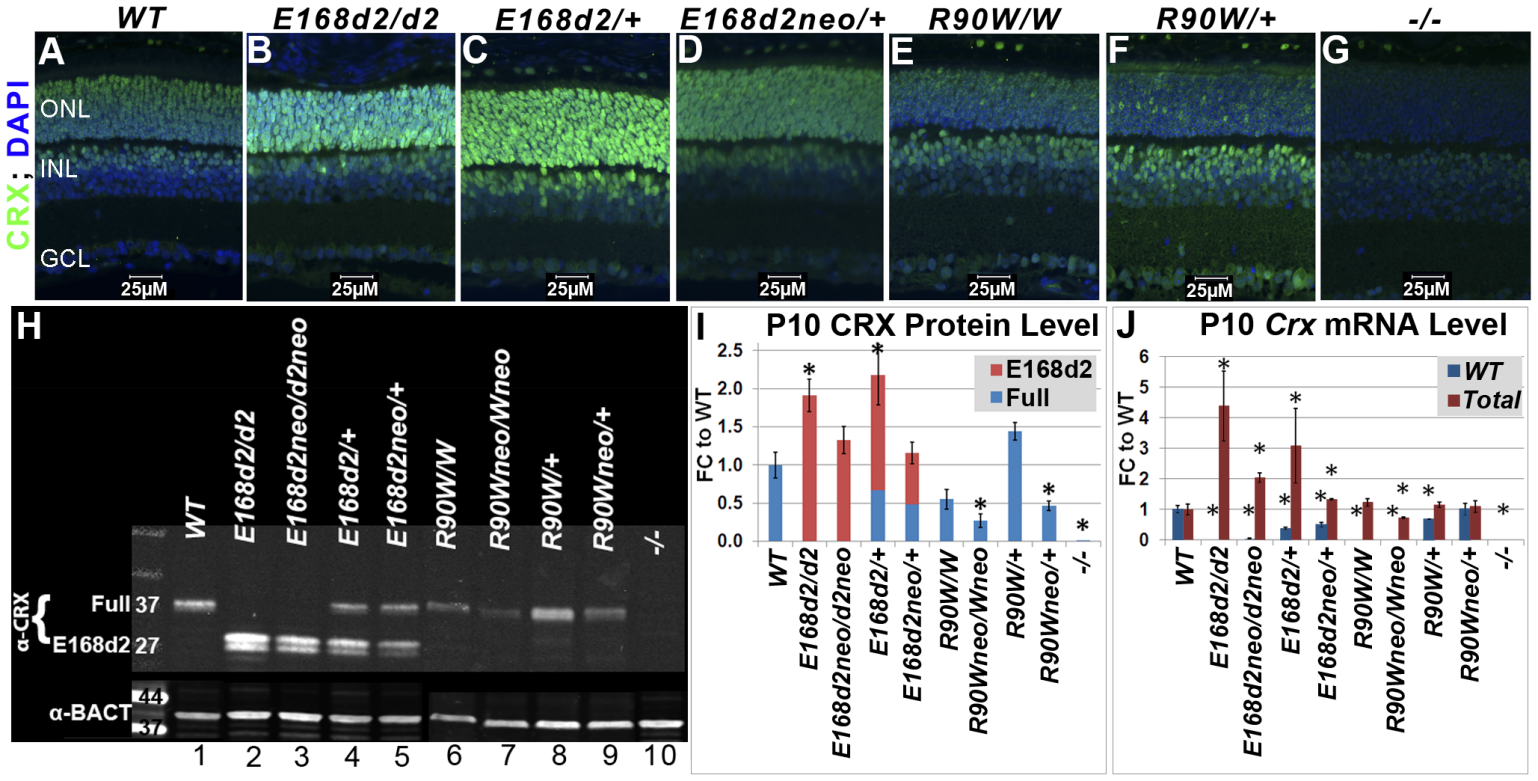
Differential expression of mutant CRX protein/RNA in *K-IN* mouse retinas. **A–G**. Paraffin embedded sagittal sections of P10 mouse retinas were stained with the mouse monoclonal CRX M02 antibody (Abnova) and imaged by fluorescent microscopy. ONL-outer nuclear layer, INL-inner nuclear layer, GCL-ganglion cell layer. **H**. SDS-PAGE and Western blot analyses of CRX proteins made by the indicated mouse strains at P10, using the rabbit polyclonal CRX 119b-1 (α-CRX) antibody [7] and mouse monoclonal anti-β-ACTIN (α-BACT, Sigma-Aldrich). Positive bands correlating with the ∼37 kD full-length CRX and ∼27 kD truncated CRX^[E168d2]^ are visible. Lanes are numbered for reference (below). **I**. CRX protein levels were quantified by measuring the intensities of the CRX^[E168d2]^ and full-length bands normalized to the β-ACTIN control using LI-COR Odyssey Image Studio software. The results are presented as fold changes (FC) relative to full-length CRX level in *WT* retina. (*p≤0.05) **J**. *Crx* mRNA levels were determined by quantitative real-time PCR using allele specific PCR primer pairs. Separate primer pairs were used to amplify *WT Crx* alone and total *Crx* (*WT*+*mutant*) in *E168d2* and *R90W* mice (see Materials and Methods). The results are presented as FC relative to *WT* retina. (*p≤0.05).

In this study, we have generated two *Crx K-IN* mouse lines, each carrying a human disease-causing mutation in the mouse allele (Accession: NM_007770.4). *Crx^E168d2^* (“*E168d2*”) mice carry a 2-bp deletion mutation, *Glu168del2*, which resulted in a codon frameshift and early truncation of the transactivation domains of CRX protein (Figure 1A–C). *Crx^R90W^* (“*R90W*”) mice carry *Arg90Trp*, an amino acid substitution mutation in the homeodomain of CRX (Figure 1A–C). An intermediate subline of each (“*E168d2neo*” and “*R90Wneo*”) carrying a *neomycin* (*neo*) cassette in intron 3–4 was also maintained (Figure 1B), since the *neo* cassette specifically reduced the expression of the mutant allele (Figure 2). The *neo* was removed from the germline by crossing *E168d2neo* and *R90Wneo* mice to the *Sox2*-*Cre* mouse [48] to generate the final *E168d2* and *R90W* mouse lines (Figure 1B). Successful *K-IN* was confirmed by PCR amplification of *neo* (Primer set: Neo F/R) and the respective *Crx* allele (Table S1, Figure S1) and Sanger sequencing of homozygous mice (Figure 1C).

### Mutant CRX is overexpressed in *E168d2* but not *R90W* mice

To determine if *E168d2* and *R90W K-IN* mice properly express their respective CRX proteins, immunofluorescence (IF) staining for CRX was performed on paraffin-embedded retinal sagittal sections of P10 mice (Figure 2). The mouse monoclonal CRX antibody M02 (Abnova) used recognizes WT (Accession: NP_031796.1) and both mutant forms of CRX. Slides were immunostained in the same batch and imaged using a common exposure. As reported previously [13] [19] [49], CRX staining in *WT* retina (Figure 2A) was predominantly localized to the outer nuclear layer (ONL), comprised of the rod and cone photoreceptor cell bodies. Less intense CRX staining was also seen in the outer portion of the inner nuclear layer (INL), which is comprised of bipolar and horizontal cell bodies. *E168d2* homozygous (“*E168d2*/*d2*”) and heterozygous (*“E168d2/+”*) mouse retinas showed higher intensity CRX staining than *WT*, especially in the ONL (Figure 2B&C). The heterozygous *E168d2neo* (*“E168d2neo/+”*) retina on the other hand showed similar intensity CRX staining as *WT* retina (Figure 2D vs 2A). In contrast, CRX staining in the ONL of *R90W* homozygous (“*R90W/W*”) and heterozygous (*“R90W/+”*) mouse retinas was reduced compared to *WT* retinas, although a few cells expressing high levels of CRX are scattered across the ONL (Figure 2E&F). This mosaic pattern of variable CRX expression was not seen in *WT* retinas. *Crx Knock-Out* (“−/−”) retinas didn't show CRX reactivity in the ONL and served as negative controls (Figure 2G). The positive CRX staining in *E168d2/d2* and *R90W/W* retinas suggests that the CRX^[E168d2]^ and CRX^[R90W]^ mutant proteins were expressed in the appropriate cell layers.

The expression levels of WT CRX and mutant CRX^[E168d2]^, CRX^[R90W]^ proteins were compared and quantified in P10 *E168d2* and *R90W K-IN* retinas using quantitative Western blots assayed with the polyclonal CRX 119b-1 antibody [7], which also recognized all forms of CRX proteins assayed. *WT* retina extracts showed a ∼37 kD band (Figure 2H, Lane 1). In contrast, a ∼27 kD dublet CRX band was detected in *E168d2/d2* (Lane 2) and homozygous *E168d2neo* (*“E168d2neo/d2neo”*) (Lane 3) retinas, suggesting that the CRX^[E168d2]^ protein was a truncated CRX protein as predicted by Sanger sequencing and genomic alignment (Figure 1C). Furthermore, the band intensities suggest that the amount of CRX^[E168d2]^ protein in mutant retinas is higher than that of the full-length CRX in *WT* retinas (Figure 2H, Lanes 2&3 vs. Lane 1). Quantification of CRX protein levels (Figure 2I) revealed a significant genotype difference (p = 0.0002) overall. *E168d2/d2* retinas made twice as much total CRX protein as *WT* retinas, while *E168d2neo/d2neo* retinas produce similar amounts of CRX protein as *WT* retinas.

Heterozygous *E168d2/+* (Figure 2H, Lane 4) and *E168d2neo/+* (Lane 5) mice expressed both full-length WT CRX and truncated CRX^[E168d2]^ protein but in different ratios. Quantification of CRX protein in *E168d2/+* retinal extracts (Figure 2I) revealed that the full-length WT CRX protein was present at approximately half of the level in *WT* retinas, but the level of CRX^[E168d2]^ protein was more than twice that of the WT CRX. As a result, the total CRX protein level in these retinas was significantly increased by 2-fold compared to normal retinas. *E168d2neo/+* retinal extracts also expressed WT CRX at approximately half *WT* levels but expressed less CRX^[E168d2]^ protein than *E168d2/+* retinas (Figure 2H, Lane 5 vs. 1&4, Figure 2I). As a result, the total CRX level in *E168d2neo/+* was comparable to the *WT* control levels. These results are consistent with immunostaining results shown in Figure 2B–D and suggest that the *E168d2* allele overproduces mutant protein, which was prevented by the presence of the *neo* cassette in *E168d2neo*.

CRX expression patterns in *R90W* mice differed from *E168d2*. In P10 *R90W/W* retinal extracts (Figure 2H, Lane 6; Figure 2I), CRX^[R90W]^ was not significantly different from CRX in *WT* retinal extracts (Figure 2H, Lane 1; Figure 2I), while levels were reduced in *R90Wneo/Wneo* retinas (Figure 2H, Lane 7; Figure 2I). *R90W/+* retinas (Figure 2H, Lane 8; Figure 2I) had normal total CRX protein levels compared to *WT* mice, although it was not possible to distinguish the quantity of WT CRX vs. CRX^[R90W]^. As seen with the *E168d2* allele, the presence of the *neo* cassette reduced total CRX protein levels in *R90Wneo/Wneo* and *R90Wneo/+* retinas, compared to corresponding *R90W* retinas (Figure 2H, Lane 7 vs. 6, Lane 9 vs. 8; Figure 2I). Thus, the presence of the *neo* cassette similarly affected the expression of both *K-IN* alleles.

To investigate whether the changes observed in CRX protein levels correlate with altered *Crx* mRNA transcription, *Crx* mRNA levels were determined by quantitative real-time reverse transcriptase PCR (qRT-PCR) (Figure 2J). Specific PCR primer pairs were used that selectively amplified sequences from either *WT* or total (*WT*+*mutant*) *Crx* cDNA (Primer sets: *Crx E168WT* F/R and *Crx R90WT* F/R; Table S1). Primer specificity was validated by amplification of *WT*, *E168d2/d2* and *R90W/W* retinal cDNA preparations. The results show that *E168d2/d2* retinas made twice as much total *Crx* mRNA as *WT* retinas, consistent with the elevated CRX protein levels in *E168d2/d2*. Total *Crx* mRNA levels in *E168d2neo/d2neo* retinas were lower than *E168d2/d2* levels (FDR p = 0.07) but remained elevated relative to the *WT* (p<0.05) retinas, in contrast to the normal total CRX protein levels observed in these retinas.

*E168d2/+* mice also showed moderately elevated total *Crx* mRNA levels (Figure 2J). Similar to protein levels, *E168d2* mRNA levels (deduced from Total - WT) were much higher than WT levels (∼2∶1 ratio). By comparison, *E168d2neo/+* mice expressed slightly elevated levels of total *Crx* mRNA that were lower than *E168d2/+*. *WT* and *E168d2* alleles were evenly expressed in these retinas. These results are consistent with the differences in CRX protein levels, supporting an RNA-based mechanism for CRX^[E168d2]^ overexpression, which was partially reversed in *E168d2neo*/+ mice.

*R90W* mice showed a distinct pattern of mRNA expression compared to *E168d2*. *R90W/W* retinas had normal *Crx* mRNA levels (Figure 2I), in contrast to their reduced CRX protein levels. This suggests a post-transcriptional mechanism either in the production or degradation of CRX^[R90W]^ protein is likely responsible. *Crx* mRNA levels in *R90Wneo/R90Wneo* mice were substantially reduced in comparison to *WT* (p<0.05) and *R90W/R90W* mice (FDR p = 0.07). The *R90W/+* and *R90Wneo/+* mice showed essentially normal levels of total *Crx* mRNA, contributed either by both alleles equally (in *R90W/+*) or the *WT* allele predominantly (in *R90Wneo/+*). Together, our results suggest that *E168d2* and *R90W* mRNA and corresponding proteins are produced in *K-IN* mouse retinas, but expression levels are differentially regulated. The mechanism of differential expression appears to be determined by features intrinsic to each mutant allele.

### Homozygous *E168d2* and *R90W* mice undergo rapid photoreceptor degeneration and are blind

**Figure 3:**
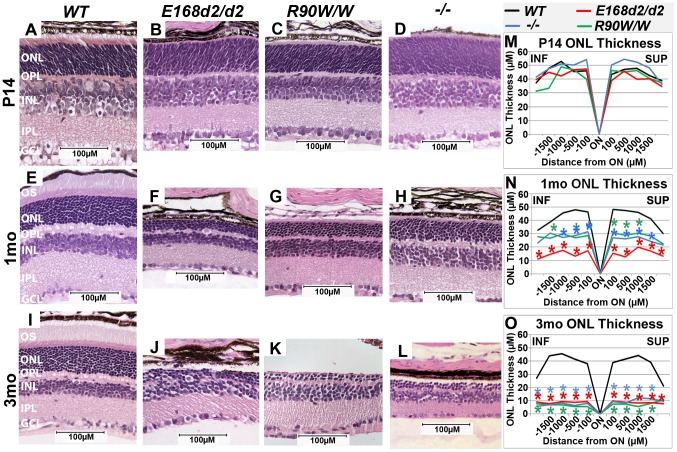
Homozygous *E168d2/d2* and *R90W/W* mice develop ‘LCA’-like retinopathy. **A–L**. H&E staining of paraffin embedded sagittal retinal sections for *E168d2/d2*, *R90W/W* and −/− mice at P14, 1 mo and 3 mo and imaged by light microscopy, showing the lack of photoreceptor outer segments (OS) and loss of ONL cells with age. **M–O**. Reduction of ONL thickness in mutant retina at each age was quantified using ‘spider graph’ morphometry. Significant differences in overall ONL thickness were determined by testing genotype*distance interactions (by two-way ANOVA) at 1 mo (p = 0.002) and 3 mo (p = 0.0001), followed by individual comparisons to *WT:* *p<0.05. INF-inferior retina, SUP-superior retina.

To determine the effect of *E168d2* and *R90W* mutations on retinal morphology, paraffin embedded retinal sections from *E168d2/d2* and *R90W/W* mice at P14, 1 month (mo) and 3 mo were stained with hematoxylin and eosin (H&E), imaged by light microscopy and compared to sections from *WT* and *−/−* mice [3] [17]. Cell specification in *WT* retina is complete by P14 and three distinct neuronal layers are present: the ONL, INL and the ganglion cell layer (GCL) (Figure 3A). At P14 *E168d2/d2*, *R90W/W* and *−/−* retinas all had established normal cellular lamination (Figure 3B–D). Quantitative morphometric measures across the sagittal plane of the retina presented by ‘spider graphs’ (Figure 3M) did not show a genotype*distance interaction (the statistical threshold required to make individual comparisons when analyzing data with two-way ANOVA) (p = 0.15) at P14. These results support previous finding that CRX is not required for retinal cell fate specification [3], including rod photoreceptors, which constitute the majority of cells in the ONL. However, unlike *WT* retinas none of the mutant ONL cells had begun to form OS's at this age (Figure 3B, C, D vs. A). This OS defect persisted through 1–3 mo when OS's were fully formed in *WT* retina (Figure 3F, G, H vs. E; J, K, L vs. I). By 1 mo, loss of ONL nuclei was evident in all mutant retinas (Figure 3F–H). In comparison to the ∼12 rows of ONL nuclei seen in *WT* retinas, *E168d2/d2* had only ∼3–4 rows, and *R90W/W* and *−/−* had ∼7–9 rows (Figure 3F, G, H vs. E). Quantification of ONL thickness shows photoreceptor degeneration occurred evenly across the sagittal plane of all mutant retinas (Figure 3N, red, green & blue lines vs. black). While *R90W/W* and *−/−* mice had similarly reduced ONL thickness (green and blue line, respectively), *E168d2/d2 retinas* showed greater ONL thinning at 1 mo (red line vs. green & blue), suggesting that degeneration was accelerated in these retinas. At 3 mo, all models exhibited greatly reduced ONL thickness (Figure 3O) with only ∼2–3 rows of ONL cells remaining (Figure 3J, K, L vs. I), suggesting ONL degeneration is progressive and extensive in all homozygous mutant mice.

To determine if ONL thinning is mediated by programmed cell death, “terminal deoxynucleotidyl transferase dUTP nick end labeling” (*TUNEL*) analysis was performed on P21 and P35 sagittal retinal sections (Figure S2). At P21 (Figure S2A–E), *E168d2/d2*, *R90W/W* and *−/−* mice all had significantly increased TUNEL+ cells present, almost exclusively in the ONL, *E168d2/d2* exhibited the highest number of TUNEL+ cells (∼34 fold over *WT*). At P35 (Figure S2F–J), TUNEL+ cells remained elevated in the ONL of all mutant models but *E168d2/d2* mice showed fewer TUNEL+ cells compared to *R90W/W* and *−/−* mice. There was no increase in TUNEL+ cells in other retinal layers of any of the mutant mice. These timecourse analyses suggest that the peak of ONL degeneration is earlier in *E168d2/d2* mice compared to *R90W/W* and *−/−* mice, corresponding with the earlier ONL thinning observed in morphometric analyses.

To assess the consequence of these morphological changes on retinal function, electroretinograms (ERG) were performed under various light intensities on *WT*, *E168d2/d2* and *R90W/W* mice at 1 month of age [50]. *E168d2/d2* and *R90W/W* mice did not show any detectable dark-adapted or light-adapted responses (Figure S3). These results suggest *E168d2/d2* and *R90W/W* mice are blind at young ages, similar to the phenotype reported for *−/−* mice [3]. The functional deficits of rod and cone photoreceptors in *E168d2/d2* and *R90W/W* mice are consistent with the necessity of photoreceptor OS's for phototransduction [15] [16] and suggest defective development of photoreceptor function in the homozygous mutant mice, similar to deficits in retinal function in LCA patients.

In spite of reduced *Crx* expression levels, homozygous mice from the sublines of each strain that carry a *neo* cassette (*E168d2neo/d2neo*, *R90Wneo/Wneo*) displayed retinal morphology and function (data not shown) that was indistinguishable from the respective *neo*-deleted line. Thus, in homozygous mice lacking *WT* alleles, the onset and rate of photoreceptor degeneration was not greatly affected by mutant protein expression level.

### Heterozygous *E168d2/+* mice, but not *R90W/+*, develop progressive rod dystrophy

**Figure 4:**
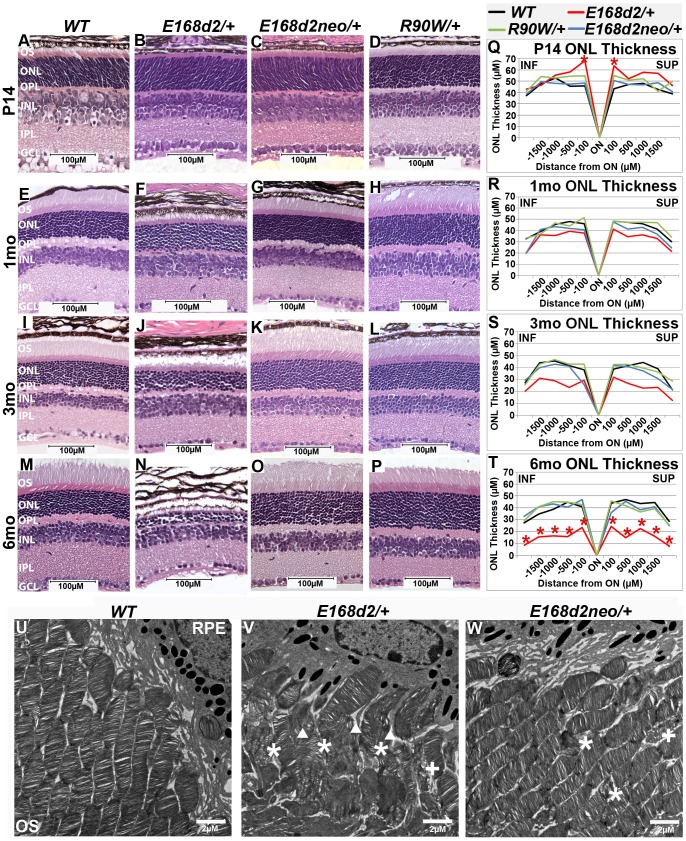
Heterozygous *E168d2/*+ mice, but not *R90W/*+, develop dominant retinopathy. **A–P**. Retinal morphology of the indicated heterozygous mutant mice was assessed by H&E staining of paraffin embedded sagittal sections at P14, 1 mo, 3 mo, and 6 mo. Shortened photoreceptor outer segments and ONL cell loss are apparent in *E168d2/+* retina only. **Q–T**. ONL thickness was assessed by spider graph morphometry at the indicated ages. *E168d2/+* (red line) shows progressive thinning of the ONL through 6 mo, while it's low expression subline, *E168d2neo/+* (blue line), and *R90W/+* (green line) do not. Significant differences in overall ONL thickness were determined by testing genotype*distance interactions (by two-way ANOVA). Significant interactions were observed at P14 (p = 0.03) and 6 mo (p = 0.03), followed by testing individual comparisons to *WT:* *p<0.05. INF-inferior retina, SUP-superior retina. **U–W**. The ultra structure of rod outer segment (OS) and nuclear morphology was assessed by transmission electron microscopy. Micrographs of rod outer segments proximal to RPE from *WT* control (**U**), *E168d2/+* (**V**) and *E168d2neo/+* mice (**W**). *E168d2/+* OS's are highly disorganized showing ‘wave-like’ OS membrane stacks (asterisks), vesiculated membranes ‘+’ and vertically oriented OS membranes (triangles). *E168d2neo/+* OS's only show minor ‘wave-like’ patterns and vesiculated membranes.

To determine the inheritance of *E168d2* and *R90W*-associated phenotypes, retinal morphology of heterozygous *E168d2/+*, *E168d2neo/+* and *R90W/+* mice was assessed by histology and morphometry. Paraffin embedded sagittal retina sections of heterozygous mutant mice at P14, 1 mo, 3 mo and 6 mo were stained with H&E, imaged by light microscopy and compared to *WT* sections (Figure 4A–P). At P14, all retinas of heterozygous mutant mice displayed normal cellular lamination (Figure 4B–D vs. A). However, morphometric measurements of the ONL thickness showed that *E168d2/+* had increased thickness at the two points most proximal to the optic nerve head (ON) (Figure 4Q, colored lines vs. black). *E168d2/+* retinas also showed shortened rod OS's compared to *WT* (Figure 4B vs. A). The OS defect in *E168d2/+* retinas remained at 1 mo (Figure 4F vs. E), 3 mo (Figure 4J vs. I) and 6 mo (Figure 4N vs. M). At 1 mo and 3 mo (Figure 4E–L, R–S), morphometric measurements of ONL thickness did not identify a significant genotype*distance interaction overall, therefore differences at each distance were not tested. However, at 3 mo, *E168d2*/+ had fewer rows of ONL cells ∼6–8 and had reduced mean ONL thickness at each distance. By 6 mo, most of *E168d2/+* ONL cells had degenerated with only ∼2–3 rows of nuclei remaining (Figure 4N vs. M; Figure 4T, red vs. black line). By morphometric analyses, *E168d2/+* exhibited reduced ONL thickness at all distances. These results suggest that *E168d2/+* retinas undergo progressive rod photoreceptor degeneration through 6 mo of age. Consistent with this observation, TUNEL analysis showed at P35 *E168d2*/+ mice had 15-fold more TUNEL+ cells than *WT* all of which were located in the ONL (Figure S2L vs. K; Figure S2O), consistent with the observed photoreceptor degeneration phenotype. These results suggest that the *E168d2* mutation causes dominant rod photoreceptor morphological defects and degeneration.

To determine if mice expressing lower levels of CRX^[E168d2]^ protein have a less severe retinal phenotype, the morphology of *E168d2neo*/+ retinas was compared with that of *E168d2*/+ retinas. At P14, similar to *E168d2*/+ (Figure 4B), the OS's of *E168d2neo/+* mice appeared shorter than in *WT* mice (Figure 4C vs. A). However, unlike *E168d2/+*, *E168d2neo/+* formed fully elongated outer segments by 1 mo (Figure 4G vs. F), which were well maintained at 3 mo (Figure 4K vs. J) and 6 mo (Figure 4O vs. M). These results suggest that, despite a delay in maturation, *E168d2neo/+* mice had less disrupted rod photoreceptor structure than *E168d2/+*. Furthermore, *E168d2neo/+* did not show significant thinning of the ONL through 6 mo (Figure 4S&T, blue vs. black line) or elevated TUNEL+ cells compared to *WT* (Figure S2M vs. K; Figure S2O). Overall, the rod photoreceptor phenotype of *E168d2neo/+* mice is mild compared to *E168d2*/+ mice, suggesting that *E168d2* disease severity was influenced by the expression level of the mutant allele in heterozygous mice, consistent with *E168d2* being an antimorphic mutation.

To further reveal morphological defects in *E168d2* photoreceptors at the ultra-structural level, transmission electron microscopy (TEM) imaging analyses were performed on the retinas of P21 *E168d2/+*, *E168d2neo/+* and *WT* mice (Figure 4U–W; Figure S4). Images were randomly coded for blinded data analysis. Compared to the morphology of *WT* OS's (Figure 4U), *E168d2/+* mice (Figure 4V) exhibited severely shortened and disordered OS's including the presence of ‘wave-like’ disc patterns (white ‘*’s), ectopic vesicle formation (white ‘+’s), and improper stacking of OS discs including vertically oriented discs (white triangles). OS morphology was largely normal in *E168d2neo/+* mice (Figure 4W); although minor ‘wave-like’ disc patterns and ectopic vesicle formation were occasionally seen.

Rod nuclei in P21 *WT* retina adopt a characteristic nuclear architecture with large areas of highly electron dense heterochromatin in the center and smaller regions of translucent euchromatin in the nuclear periphery [51] (Figure S4A&D). The chromatin pattern of *E168d2/+* rods, however, appeared less condensed than *WT* (Figure S4B&E vs. A&D). This did not occur in *E168d2neo/+* mice (Figure S4C&F vs. A&D). To quantify these changes, the percentage of the nuclear area comprised of condensed heterochromatin was measured in randomly selected *WT*, *E168d2/+* and *E168d2neo/+* rod nuclei. Figure S4G shows that the mean area of heterochromatin in *E168d2/+* rods was significantly reduced by 8% compared to *WT*. This reduction in rod heterochromatin territory was not seen in *E168d2neo/+* mice, suggesting more normal rod nuclear architecture. In addition, photoreceptor degeneration in *E168d2*/+ and *E168d2neo/+* mice was evidenced by the presence of highly electron dense nuclei corresponding to pyknotic photoreceptor cells undergoing cell death, which were not observed in *WT* retinas (Figure S4E&F vs. G, white pentagon).

Unlike *E168d2/+*, *R90W/+* mice had normal retinal morphology at all ages (Figure 4D, H, L&P), comparable to *+/−* mice [3]. They formed and maintained full-length OS's and normal ONL thickness (Figure 4H, L&P) through 6 mo of age. No increase in TUNEL+ cells over *WT* was detected (Figure S2N&O). These results suggest rod photoreceptor development and maintenance are normal in *R90W/+* mice. This is consistent with clinical evaluations for heterozygous *R90W* carriers in human cases [22] [23] [33] [42] [45].

### Heterozygous *E168d2/+* mice develop early-onset cone dystrophy

#### Mislocalization of cone nuclei

Cone photoreceptors comprise only ∼3% of ONL cells in mouse retina and their integrity could not be accurately assessed by light microscopy-based histology alone. However, cone nuclei were identified in TEM micrographs by their distinct decondensed chromatin patterns [51] and their nuclear position near the outer edge of the ONL (Figure S4A, white arrows). In the retinas of P21 *E168d2*/+ mice, few cone nuclei were identifiable in the ONL (Figure S4B&E). The majority of nuclei with ‘cone-like’ decondensed chromatin were misplaced to the inner regions of the ONL adjacent to the OPL (Figure S4E, white arrows).

The number of identifiable cone nuclei in *E168d2neo*/+ mice was greatly increased compared to *E168d2*/+, but nuclei were frequently mislocalized to the middle and inner ONL (Figure S4F, white arrows). Thus, cone formation/survival is improved in *E168d2neo*/+ mice but cone nuclear localization remains abnormal. Taken together, the ultra-structural analyses suggest that rod and cone photoreceptor morphology is highly disrupted in *E168d2/+* mice, and less so in *E168d2neo*/+ mice.

**Figure 5:**
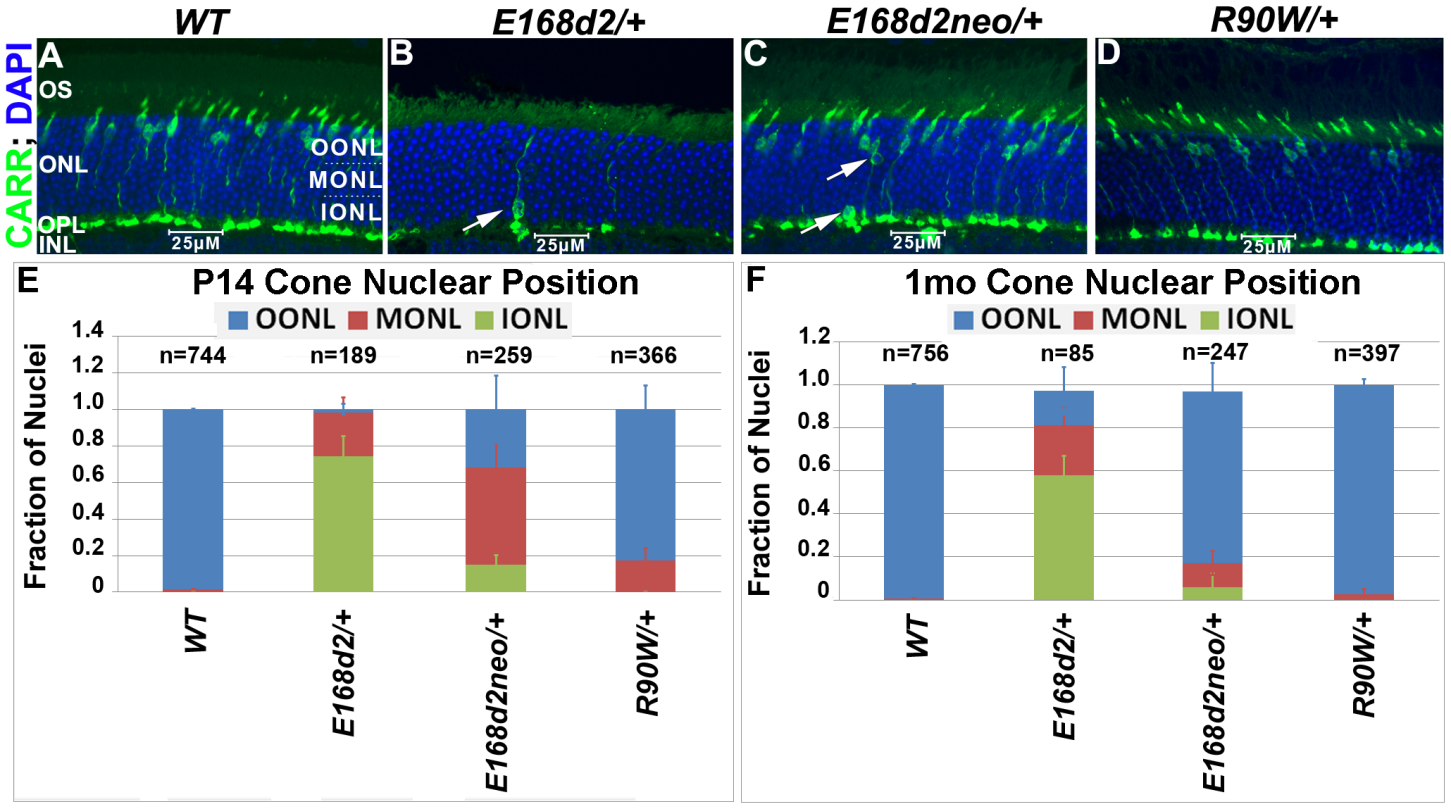
Heterozygous *E168d2/*+, *E168d2neo/*+ and *R90W/*+ mice display abnormal cone nuclear localization in developing and adult retina. **A–D**. Sagittal retinal sections from the indicated mice at 1 mo, stained for cone arrestin (CARR) (green) and nuclear marker DAPI (blue). To assess cone nuclear location, the ONL was arbitrarily divided into three zones, outer ONL (OONL), mid ONL (MONL) and inner ONL (IONL). **A**. *WT* cone nuclei were found only in the OONL, while *E168d2/+*, *E168d2neo/+* and *R90W/+* had varying numbers of cones localized to IONL or MONL (**B**&**C** white arrows). **E, F**. Cone nuclear position was quantified by counting the fraction of CARR+ nuclei in each ONL zone of sagittal retinal sections from P14 or 1 mo mice of the indicated genotype (*p<0.05).

To determine whether the scattered nuclei with decondensed ‘cone-like’ chromatin in *E168d2/+* and *E168d2neo/+* were indicative of mislocalized cone nuclei, cone specific markers were used to further assess the cell population. Paraffin-embedded retinal sections were immunostained for cone arrestin (CARR, Accession: Q9EQP6.1) (antibody: rabbit polyclonal α-mCARR, Millipore), which stained the cone cell body from the inner segment to the synaptic terminal (Figure 5A–D). Normal cones undergo nuclear migration during development, reaching their final position at the apical ONL by P12 [52] [53]. Retinal sagittal sections immunostained with CARR were analyzed to determine cone nuclear position in *WT* and mutant retinas (Figure 5A–D). Nuclei were assigned to three zones: Inner (IONL), Mid (MONL) or Outer ONL (OONL) (Figure 5A). At P14, while most *WT* cone nuclei were positioned in the OONL, cone nuclei in *E168d2/+* were mostly (>70%) positioned in the IONL and the majority of E168d2neo/+ cone nuclei (∼70%) were mislocalized in the MONL (Figure 5E, Figure S5A). At 1 mo, *E168d2*/+ cone nuclei remained highly scattered (Figure 5B, white arrow) with less than 20% localized to OONL (Figure 5F), while most *E168d2neo*/+ cone nuclei (∼80%) had migrated to the OONL although a significant number (11%) remained in the MONL (Figure 5C white arrows; Figure 5F; Figure S5B). Thus, cone nuclear migration was largely ablated in *E168d2*/+ mice, while this phenotype was less severe in *E168d2neo*/+ mice. In contrast, cone nuclei localization in *R90W*/+ retina was mildly affected at P14 (∼17% in MONL) (Figure 5E) but was normal at 1 mo of age (Figure 5D&F).

#### Progressive cone degeneration

**Figure 6:**
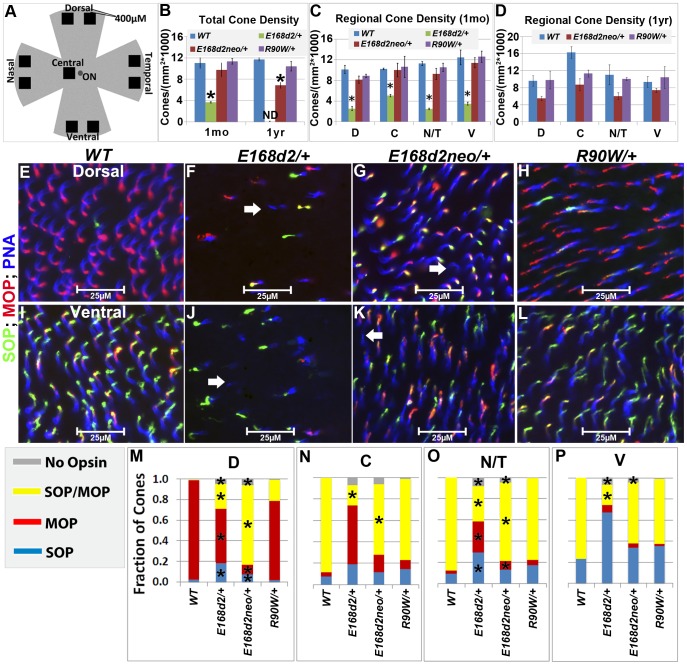
Heterozygous *E168d2/*+, *E168d2neo/*+ and *R90W/*+ mice display distinct changes in cone density and M/S opsin gradient formation. **A**. Diagram showing regions of flat-mounted retina selected for cone density image analyses. **B–D**. Cone density of 1 mo and 1 yr old mice was determined by counting PNA+ cells on flat-mounted retinas in the dorsal (D), central (C), nasal/temporal (N/T) and ventral (V) regions. **B**. Total cone density over all regions (*p<0.05). **C–D**. Cone density in each region in1 mo (**C**, *p<0.05) and 1 yr old (**D**) mice. ND-not determined. Error bars: SEM. Note that genotype*retinal region interaction (by two-way ANOVA) was significant at 1 mo (p = 0.04) but not 1 yr (p = 0.11). **E–L**. Flat-mounted retinas from 1 mo mice of the indicated genotype were stained for OPN1SW (SOP, green), red/green opsin (MOP, red) and the pan cone marker peanut agglutinin (PNA, blue), showing sample images from the dorsal (**E–H**) and ventral (**I–L**) regions. Image scale bars: 25 µM. Unlike *WT* samples (**E&I**), *E168d2/+*, (**F&J**) and *E168d2neo/+* (**G&K**) samples show a small number of PNA+ cones that did not express either cone opsin (white arrows). **M–P**. Fraction of cones in each region expressing SOP, MOP, both SOP/MOP or no opsin (*p<0.05).

The numbers of CARR+ cones in P14 and 1 mo *E168d2/+* retina sections were noticeably reduced compared to *WT* retina (Figure 5B). This could have been caused by either missing cone photoreceptors or aberrant CARR expression. Indeed, the expression of CARR was previously shown to be CRX-dependent [3] and was reduced in *E168d2/+* and *E168d2neo*/+ retinas (see below). To accurately determine the integrity of the cone population in heterozygous mutant retinas, another pan cone marker, peanut agglutinin conjugated to Rhodamine (PNA, Vector labs) was used in immunofluorescence staining of whole-mount retinas (Figure 6). Unlike CARR, PNA reactivity was independent of CRX's regulatory function and marked the membrane sheath of all cones [54], thus allowing for the accurate assessment of cone density in mutant retinas. Whole-mount retina preparations of 1 mo and 1 year (yr) old heterozygous K-IN mice were stained with PNA (Figure 6E–L, blue stain). 40× fluorescent images were taken of the dorsal (D), ventral (V), nasal/temporal (N/T) and central (C) retina (diagrammed in Figure 6A), and cone density from each region was determined by counting PNA+ cells. At 1 mo, total cone density from *E168d2*/+ retinas over all regions was reduced by 67.7±1.3% (Figure 6B&C, green vs. blue bars), suggesting a cone deficit prior to rod degeneration. Cone density in 1 yr old *E168d2/+* retinas was not determined because ONL degeneration was already extensive by 6 mo (Figure 4N). Cone density was preserved in *E168d2neo*/+ mice at 1 mo but was reduced by 39.6±5.3% at 1 yr (Figure 6B, red vs. blue bar in each age group). Further comparing the cone density in different regions of *E168d2neo*/+ retina showed that cone density was normal in all regions at 1 mo (Figure 6C, red vs. blue bars) and in the ventral retina at 1 yr of age, but was reduced in all other regions (Figure 6D, red vs. blue bars). These results suggest that *E168d2*/+ mice had early cone deficits, while cones were maintained longer in *E168d2neo*/+ retinas. In contrast, *R90W*/+ mice had normal overall cone density through 1 yr (Figure 6B&C, purple vs. blue bars), despite modestly reduced cone density in the central region with age.

#### Malformation of M/S cone opsin gradient

Mouse cones consist of three subtypes defined by which cone opsins they express: OPN1MW (MOP, Accession: NP_032132.1), OPN1SW (SOP, Accession: NP_031564.1) or both opsins. In normal mouse retina, MOP and SOP are expressed in opposing gradients along the dorsal-ventral axis [55]. In the dorsal retina, a high percentage of cones express MOP, a few cones express SOP and no cones express both. Moving towards the central and ventral retina, there is an increase in SOP and MOP/SOP co-expressing cones [56]. The formation of the cone opsin gradient in *Crx* mutant retinas was assessed by IF staining of whole-mount retinas with polyclonal rabbit anti-red/green opsin (Millipore), polyclonal goat anti-OPN1SW (Santa Cruz) antibodies and PNA. Fluorescence images acquired in the regions diagrammed in Figure 6A of control *WT* retinas showed the clear formation of the cone opsin gradient as expected (Figure 6E&I).

At 1 mo, *E168d2/+* mice had low levels of the cone opsins in their outer segments and did not establish the M/S opsin gradient properly (Figure 6F&J). This conclusion was confirmed by quantification of the fraction of cones (PNA+) expressing MOP, SOP, both opsins or no opsin in tested regions (Figure 6M–P). *E168d2/+* dorsal retina showed a reduction in the proportion of cones expressing MOP only, but an increase in cones expressing SOP or both opsins (Figure 6F&M, *E168d2/+* vs. *WT*). In contrast, *E168d2/+* ventral retina showed a large decrease in the percentage of MOP/SOP co-expressing cones and an increase in cones expressing SOP only (Figure 6J&P, *E168d2/+* vs. *WT*). Changes in M/S opsin patterns in central and nasal/temporal regions were also seen in *E168d2/+* mice (Figure 6N&O, *E168d2/+* vs. *WT*). Overall, *E168d2*/+ mice had a lower percentage of co-expressing cones and failed to properly regulate opsin expression across the dorsal-ventral axis. In addition, the levels of opsin on individual cone outer segments were highly variable in *E168d2/+* retinas, and some PNA+ cells did not have any detectable opsin (Figure 6F&J, white arrows). These results suggest that cone opsin expression, trafficking, or both were affected in *E168d2/+* retina.

In contrast, the levels of MOP and SOP were closer to normal in *E168d2neo*/+ cones but the opsin gradient remained highly disrupted (Figure 6G&K vs. E&I). As shown in the bar graphs, the percentage of cones co-expressing MOP/SOP was increased dramatically in *E168d2neo*/+ dorsal retina (Figure 6M, *E168d2neo/+* vs. *WT*), while the percentage of co-expressing cones was decreased in the central, nasal/temporal and ventral retina (Figure 6N–P, *E168d2neo/+* vs. *WT*). These results suggested that in *E168d2neo*/+ retinas, despite having normal cone numbers at 1 mo, the cone opsin gradient was not properly established, which may have contributed to the deficits in cone function (see below) and long-term survival.

By comparison, *R90W/+* mice showed largely normal M/S opsin expression and gradient formation across the dorsal to ventral retina (Figure 6H&L vs. E&I). Quantification did not reveal any significant differences in the fraction of cones expressing each opsin in any of the regions surveyed (Figure 6M–P, *R90W/+* vs. *WT*).

Taken together, assessment of the cone photoreceptor population in mutant mice reveals that cones do not develop properly in *E168d2/+* retinas and cone defects arise earlier and are more severe than rod defects. *E168d2neo*/+ retinas showed more normal cone photoreceptor development and morphology and slower cone degeneration than *E168d2*/+ retinas. However, cone subtype specification remained disrupted in *E168d2neo*/+ retinas. Reduction in the number of cones in *E168d2*/+ and *E168d2neo*/+ before rod degeneration was consistent with a ‘cone-centric’ phenotype. In contrast, *R90W/+* mice had largely normal cone morphology, did not exhibit any significant cone subtype differences and no changes in overall cone density through 1 yr.

### Functional defects in heterozygous *E168d2/+* and *R90W/+* mice

#### Heterozygous *E168d2* mice show severe rod/cone functional deficits

**Figure 7:**
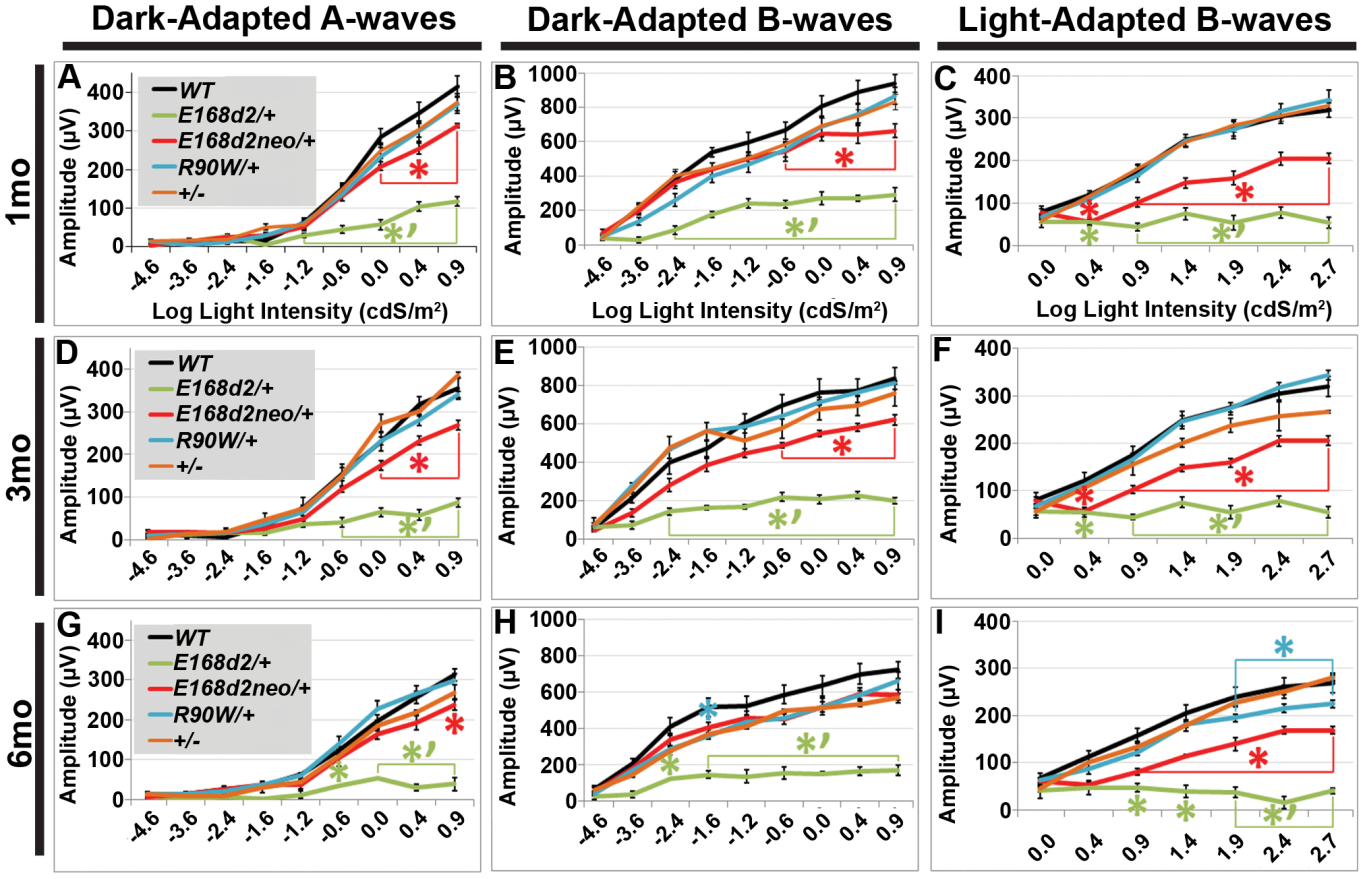
Heterozygous *E168d2/*+, *E168d2neo/*+ and *R90W/*+ mice have graded deficits in retinal function. **A–I**. Retinal function of *E168d2/+*, *E168d2neo/+* and *R90W/+* and *+/−* mice was assessed by electroretinography at 1 mo (**A–C**), 3 mo (**D–F**) and 6 mo (**G–I**). Average peak amplitude responses for dark-adapted A-waves and B-waves and light-adapted B-waves are plotted against the log of the flash intensity (Log [cd*s/m^2^]). Genotype*flash intensity interactions for peak amplitude (by two-way ANOVA) were significant (p<0.05) at all ages for each wave form tested. *E168d2/+* mice show severe deficits in all wave responses at each age compared to responses from either *WT* or *E168d2neo/+* mice (green vs. black and red line, respectively). Peak responses in *E168d2neo/+* mice are higher than *E168d2/+* (red vs. green line), but remain significantly decreased compared to *WT* (red vs. black line) with exceptions for 6 mo dark-adapted B-waves (**H**). *R90W/+* and *+/−* mice have mostly normal retinal function (blue or orange vs. black line) but *R90W/+* have subtle significant deficits in light-adapted B-waves at 6 mo (**I**, blue vs. black line). (Relative to *WT*: *p<0.05; relative to *E168d2neo/+*: *‘p<0.05, brackets indicate all enclosed data points are significant). Error bars: SEM.

To determine if rod and cone photoreceptor morphological abnormalities and degeneration correspond with impaired retinal function, ERG's were performed on *E168d2/+*, *E168d2neo*/+, *R90W/+*, and *+/−* mice at 1 mo, 3 mo and 6 mo (Figure 7). First, ERG analyses were carried out on dark-adapted animals to assess rod-driven function. The responses to light flashes of increasing intensities were recorded, and the amplitudes of the A-waves (arising from the hyperpolarization of photoreceptors) and B-waves (arising from the activity of the photoreceptor-driven inner retina) [50] were measured. The results were plotted as average peak amplitudes of A and B-waves, against serial log scale light intensities (Figure 7A, B, D, E, G&H, black line). Next, ERGs were performed after 10 minutes of light adaptation to measure cone-driven responses. The average peak amplitudes of light-adapted B-waves were plotted against log scale light intensity (Figure 7C, F&I, black line). A significant genotype*light flash intensity interaction (by two-way ANOVA, p<0.05) was detected at every time point for both dark and light-adapted tests. At 1 mo, both dark-adapted A and B-waves were detectable in *E168d2/+* mice particularly in high light intensities, but the peak amplitudes were significantly reduced compared to *WT* controls (Figure 7A&B, green vs. black line), indicating impaired “rod-driven” function. *E168d2/+* rod function declined further with age, as the peak amplitudes became progressively smaller at 3 mo and 6 mo (Figure 7D, G vs. A; E, H vs. B, green line), corresponding with rod degeneration. For illustrative purposes, the progressive nature of rod functional deficits was demonstrated by the mean percent reductions of dark-adapted A and B-wave amplitudes (Table 1, *E168d2/+* columns). Percent reduction for both A and B-waves increased from 1 mo to 6 mo suggesting further deviation from *WT* function.

**Table 1:** Percent reduction* of ERG peak amplitudes in *E168d2/+* and *E168d2neo/+* mice.

	Dark-adapted A -waves Average: −0.02, 0.387, 0.875 Cds/m^2^ flashes		Dark-adapted B-waves Average: −0.02, 0.387, 0.875 Cds/m^2^ flashes		Light-adapted B-waves Average: 0.40, 0.88, 1.38, 1.88, 2.39, 2.82 Cds/m^2^ flashes	
Age	*E168d2/*+	*E168d2neo/*+	*E168d2/*+	*E168d2neo/*+	*E168d2/*+	*E168d2neo/*+
1 mo	73.3±5.3	25.9±1.1	68.2±1.6	25.6±5.3	72.8±10.2	41.3±7.3
3 mo	76.4±5.1	25.2±1.2	73.1±2.8	26.3±1.8	82.4±4.3	46.7±6.3
6 mo	82.6±8.7	21.8±4.5	76.3±0.3	17.3±1.9	78.9±12.9	41.4±5.3

* Average percent reductions were calculated based on Figure 7 data. ±STDEV.

Cone-driven light-adapted B-wave peak amplitudes were barely detectable in *E168d2/+* at 1 mo (Figure 7C, green line) and all later ages tested (Figure 7F&I, green line), corresponding with the early reduction of cone number. The mean percent reductions in light-adapted B-wave amplitudes were more severe than those seen in dark-adapted A or B-wave amplitudes (Table 1, *E168d2/+* columns), suggesting that cone function was more severely affected than rod function in *E168d2/+* mice.

Compared to *E168d2/+*, *E168d2neo*/+ mice show significantly less impaired dark-adapted A and B-wave peak amplitudes for most light intensities at all the ages tested (Figure 7A, B, D, E, G&H, red line vs. green line). *E168d2neo*/+ mice only had minor ‘rod-driven’ functional deficits compared to *WT* mice (red line vs. black line). These findings are summarized in Table 1. The average percent reductions of the dark-adapted A and B-waves in *E168d2neo*/+ mice were much less than *E168d2/+* for all three ages tested. More importantly, the minor deficits in *E168d2neo*/+ ‘rod-driven’ function did not progress with age, consistent with improved rod survival. Light-adapted B-waves were also significantly more robust in *E168d2neo*/+ mice, compared to *E168d2*/+ mice (Figure 7C, F&I, red vs. green line), but remained significantly reduced, compared to *WT* mice (red vs. black line). *E168d2neo*/+ cone deficits were more severe than rod deficits as shown by higher percent reductions in light-adapted B-waves than dark-adapted B-waves (Table 1, *E168d2neo*/+ columns). These defects were first detected at 1 mo and persist through 6 mo. Thus, while *E168d2*/+ mice had severely impaired rod and cone function resembling an ‘LCA’ phenotype, *E168d2neo*/+ mice had ‘cone-centric’ deficits in retinal function, modeling a ‘CoRD’ phenotype. The ‘cone-centric’ morphological and functional deficits of *E168d2neo*/+ mice, together with the early cone deficits in *E168d2*/+ mice, suggest that cones may be more sensitive than rods to the antimorphic effect of CRX^[E168d2]^ protein.

#### Heterozygous *R90W* mice show minor late-onset cone functional deficits

Previous studies report subtle ERG deficits in +/− mice [3], but in our studies *R90W/+* and +/− mice did not show significant ERG deficits at 1 mo or 3 mo (Figure 7A–F, blue and orange line, respectively). At 6 mo, *R90W*/+ mice exhibit minor light-adapted B-wave deficits at the 1.88, 2.39 and 2.82 cdS/m^2^ flash intensities (Figure 7I, blue vs. black line), while +/− were functionally normal, suggesting late-stage cone defects in *R90W*/+ mice. The difference between our studies and previous studies on +/− could have been due to mouse strain background, since the original *Crx KO* characterization was performed on a mixed background of *129Sv*×*C57BL/6* [3]. All experiments in this paper were performed on a congenic *C57BL/6J* background. Nevertheless, the *R90W* mutation produced only a mild late-stage cone functional phenotype in heterozygous mice, while the *E168d2* mutation in heterozygous mice caused an early-onset severe impairment of rod and cone function that depended on the expression level of the *E168d2* allele relative to *WT*.

### *E168d2* mutation impairs target gene expression more severely than null, while *R90W* mutation produces a hypomorphic effect

#### Gene expression changes in homozygous mutants

**Figure 8:**
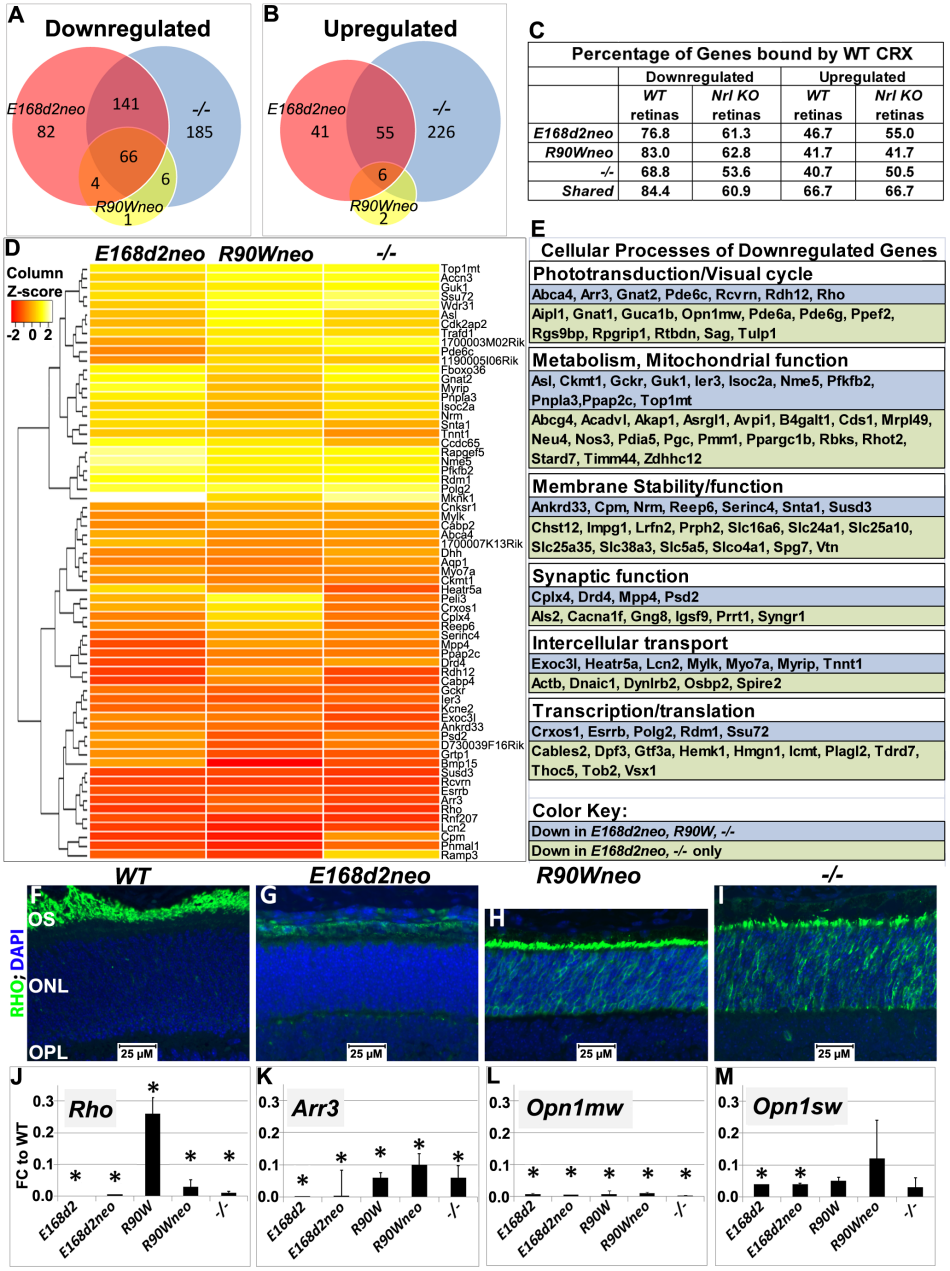
Homozygous *E168d2*, *R90W* and −/− mice show graded changes in retinal gene expression. **A–B**. Venn diagram showing overlap of genes that are differentially expressed at P10, as identified by Illumina gene expression mouseRef6 microarray. The number of genes in each group is indicated. *E168d2neo/d2neo*, *R90Wneo/Wneo* and *−/−* mice show a high degree of overlap in differentially expressed genes. **C**. Percentage of differentially expressed genes for each genotype that are directly bound by WT CRX protein in *WT* and *Nrl KO* retinas (based on the published ChIP-Seq datasets [21]). For all mutant genotypes, differentially expressed genes are enriched for direct CRX targets. **D**. Heat map of commonly downregulated genes in *E168d2neo/d2neo*, *R90Wneo/Wneo* and *−/−* mice show graded changes in gene expression of commonly downregulated genes. **E**. Cellular processes associated with commonly downregulated genes, based on gene ontology provided by Mouse Genome Informatics, showing a widespread effect of *Crx* mutations on visual and cellular pathways. **F–I**. P14 paraffin embedded sagittal retinal sections of *WT*, *E168d2neo/d2neo*, *R90Wneo/Wneo and −/−* mice were stained with Rhodopsin (RHO, green) and DAPI (blue), and imaged by wide field fluorescence at 40×. Note that RHO is absent in *E168d2neo/d2neo*, while mislocalized to ONL in *R90Wneo/Wneo* and *−/−*. **J–M**. Validation of microarray results by qRT-PCR analyses on selected CRX target genes, *Rho*, *Arr3*, *Opn1mw* and *Opn1sw* in retinas of P10 homozygous mice from the indicated strains, shown as FC relative to *WT*. (*p<0.05; Error bars: STDEV).

To understand the molecular mechanisms underlying the observed morphological and functional deficits in *K-IN* mutant retinas, we compared retinal transcription profiles of homozygous *E168d2neo* and *R90Wneo* mice with *WT* and −/− controls to determine the effects of mutant CRX protein on target gene expression. We chose to use homozygous mice from the *neo*+ sublines, which express the mutant protein at lower levels than the final lines, for the gene profiling analyses to avoid alterations in gene expression that could arise strictly from mutant CRX overexpression. Unlike subline differences observed in heterozygous mutant mice, morphological and functional characterization demonstrated that the phenotypes of homozygous mice from *neo+* and final *neo*- sublines were indistinguishable. Expression profiling results were validated by qRT-PCR for several genes in all sublines. RNA from sex-matched pairs of P10 retinas for each genotype was reverse transcribed into cDNA, which was hybridized onto Illumina mouse Ref6 expression microarrays. Microarray analyses showed a high degree of overlap of differentially expressed genes in homozygous *E168d2neo*, *R90Wneo* and *−/−* mice (Figure 8A&B). The complete list of differentially expressed genes is available in Tables S2 and Table S3, and the raw datasets are available at NCBI GEO website (http://www.ncbi.nlm.nih.gov/gds, access number: GSE51184). For downregulated genes: 70.6% of *E168d2neo* and 93.5% of *R90Wneo* genes were shared with *−/−* (Figure 8A). For upregulated genes: 59.8% of *E168d2neo* and 75% of *R90Wneo* genes were shared with *−/−* (Figure 8B).

To determine which differentially expressed genes were directly bound by CRX, datasets from chromatin immunoprecipitation sequencing (ChIP-Seq) of WT CRX protein in *WT* and *Nrl Knock-Out* mice [21] were compared with target genes identified by microarray. Downregulated genes in *E168d2neo*, *R90Wneo* and *−/−* mice showed a high correlation of direct CRX targets (76.8% *E168d2neo*, 83.0% *R90Wneo*, 84.37% of shared genes), compared to upregulated genes (46.7% *E168d2neo*, 41.7% *R90Wneo*, 66.7% of shared genes) suggesting that mutation or loss of CRX is mainly associated with reduced expression of direct CRX target genes (Figure 8C). However, upregulated genes were more frequently bound by CRX than binding genome-wide (22.6%), suggesting that mutation or loss of CRX also affects expression of genes directly repressed by CRX.

While *E168d2neo*, *R90Wneo* and *−/−* mutations affected similar sets of genes, distinct degrees of gene expression changes were observed. Heat map analysis showed the majority of shared downregulated genes were more strongly reduced in *E168d2neo* and *−/−* compared to *R90Wneo* (Figure 8D). The less reduced expression of downregulated CRX target genes in *R90Wneo* mice suggests that *R90Wneo* retains some weak ability to promote transcription. *E168d2neo* and *−/−* had similar heat map profiles but several key photoreceptor genes were lower in *E168d2neo* including: *Rho*, *Arr3*, *Ramp3*, *Drd4*, *Cpm*, and *Pde6c* (Accessions: NM_145383.1, NM_133205.3, NM_019511.3, NM_007878.2, NM_027468.1, AF411063.1, respectively). The more severe reduction in gene expression in *E168d2* is consistent with its accelerated photoreceptor degeneration compared to *R90W* and *−/−*. It is notable that many shared downregulated genes encode proteins in the phototransduction and visual cycle pathways essential for establishing vision (Figure 8E), including: *Opn1sw*, *Opn1mw*, *Gnat1*, *Gnat2*, *Rcvrn*, *Pde6a*, *Pde6g*, (Accessions: NM_007538.3, NM_008106.2, NM_008140.2, NM_008141.2, NM_009038.2, NM_146086.2, NM_012065.2, respectively) *Rho*, *Arr3*, and *Pde6c*. Other downregulated genes encode proteins with function in key cellular processes, including metabolism and mitochondrial function, membrane stability/function, synaptic function, intercellular transport, and transcription/translation (Figure 8E), which likely contribute to the morphological and functional defects. Furthermore, many downregulated genes were associated with the human diseases RP, LCA and CoRD (Table S4, https://sph.uth.edu/retnet/), overlapping with those reported for *CRX* mutations.

To confirm graded changes in target gene expression, levels of several key CRX target genes were validated in homozygous mice of all mutant lines using IF staining (Figure 8F–I) and/or qRT-PCR analyses (Figure 8J–M, Table S5, Table S6). IF staining showed that homozygous *E168d2neo* retinas failed to produce rhodopsin (RHO) in contrast to *−/−* which still made a low amount of RHO (Figure 8G vs. I). This suggests that CRX^[E168d2]^ protein has an inhibitory effect on *Rho* expression beyond complete loss of CRX protein. Homozygous *R90Wneo* retinas, on the other hand, produced RHO at a level higher than *−/−* but much lower than *WT* retinas (Figure 8H vs. F&I). Expression changes of a number of genes in the retinas of homozygous mutants from the *neo+* and final *neo*- lines were validated using qRT-PCR, including the rod gene *Rho* and the cone genes *Arr3*, *Opn1sw* and *Opn1mw* (Figure 8J–M, Table S5). The results, presented as fold change (FC) relative to *WT*, from P10 homozygous mice of the ‘low expression’ subline and final line of each mutation were compared (Figure 8J–M). *Rho* expression was essentially abolished in both *E168d2* and *E168d2neo* mice. In contrast, *R90W* and *R90Wneo* mice expressed *Rho* at levels slightly higher than *−/−* (Figure 8J). These results are consistent with the microarray and IF results described above. Expression of the cone gene *Arr3* was not detectable in *E168d2* or *E168d2neo* mice, while residual amounts were detected in *R90W*, *R90Wneo* and *−/−* mice (Figure 8K). *Opn1sw* and *Opn1mw* were strongly downregulated in homozygous mice of all models. The loss of expression of genes involved in rod/cone phototransduction explains the loss of visual function in these mice. Together, our results suggest that the *E168d2* mutation produced a direct antimorphic effect on photoreceptor gene expression beyond CRX deficiency, while *R90W* is a hypomorph mutation, resulting in a CRX protein with impaired residual transcriptional regulatory function.

#### Gene expression changes in heterozygous mutants

**Figure 9:**
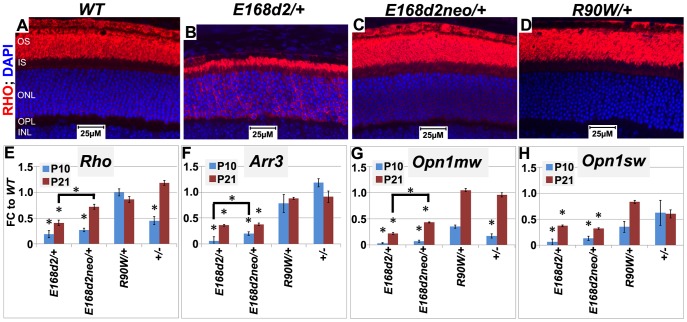
Graded changes in CRX target gene expression in heterozygous *E168d2/*+, *E168d2neo/*+ and *R90W/*+ mice. **A–D**. Paraffin embedded sagittal retinal sections of 1 mo *WT* and the indicated heterozygous mutant mice were stained with mouse monoclonal anti-Rhodopsin RetP-1 antibody (Chemicon) (RHO, red) and DAPI nuclear conterstaining (blue), and imaged by widefield fluorescence at 40×. *E168d2/+* shows reduced rod OS length and mislocalized RHO in ONL. **E–H**. qRT-PCR analysis of four CRX target genes, *Rho*, *Arr3*, *Opn1mw*, *Opn1sw* in the indicated heterozygous mice at P10 and P21 (*p<0.05; bracketed *FDR p<0.09; Error bars: SEM). Note at P10, the expression of *Opn1mw* and *Opn1sw* CRX target genes are reduced in all mutant models. However, at P21, expression recovers in *R90W/+* and *+/−* mice, while remaining reduced in *E168d2/+* and *E168d2neo/+* mice.

To determine if gene expression changes in heterozygous mice followed the same trend as homozygous mutants, expression of the selected CRX target genes, *Rho*, *Arr3*, *Opn1sw* and *Opn1mw*, was evaluated in heterozygous mutants using IF staining of retinal sections at 1 mo of age (Figure 9A–D) and/or qRT-PCR at P10 and P21 (Figure 9E–H, Table S5, Table S6). IF staining for RHO at 1 mo showed that *E168d2/+* mice displayed low intensity of RHO staining, some of which was mislocalized to the ONL (Figure 9B vs. A), suggesting impaired RHO trafficking. This phenotype was not seen in *E168d2neo/+* and *R90W/+* retinas (Figure 9C, D vs. B). qRT-PCR analyses showed that *E168d2/+* mice exhibited persistent downregulation of all genes tested in both P10 and P21, despite some degree of recovery at P21 (Figure 9E–H, Table S5, Table S6). Expression of these genes was also decreased in *E168d2neo/+* mice, but significant improvements were seen for *Rho* and *Opn1mw*, compared to *E168d2*/+ mice. The more severe impairment of gene expression in *E168d2/+* compared to *E168d2neo/+* demonstrates the dosage effect of CRX^[E168d2]^ mutant protein. In contrast, *R90W/+* and +/− mice exhibited reduction in the expression of some genes at P10 but normal expression was observed at P21. The degree of expression changes in individual mutants varied from gene to gene. These results suggest that CRX^[E168d2]^ mutant protein actively impaired CRX target gene transcription in the presence of WT protein, consistent with an antimorphic mechanism. This antimorphic effect depends on CRX^[E168d2]^ protein dosage and can be reduced by decreasing CRX^[E168d2]^ expression. In contrast, *R90W/+* essentially phenocopied the phenotype of +/−, suggesting that CRX^[R90W]^ protein did not severely interfere with the function of WT CRX *in vivo*, which allowed for normal photoreceptor gene expression by P21.

### CRX^[E168d2]^ protein binds target DNA and interferes with WT CRX function, while CRX^[R90W]^ protein retains marginal DNA binding and transactivating activity

**Figure 10:**
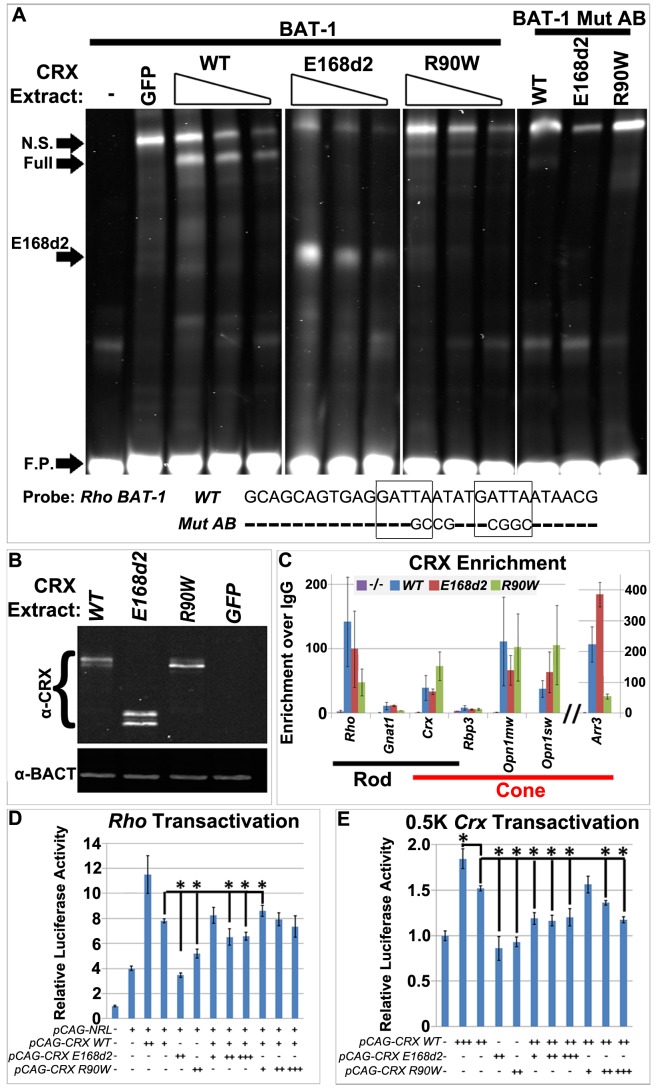
CRX^[E168d2]^ and CRX^[R90W]^ affect target gene transcription through distinct molecular mechanisms. **A**. Electrophoresis mobility shift assays (EMSA) to measure the DNA binding activity of HEK293-expressed CRX^[E168d2]^ and CRX^[R90W]^ protein to the *Rho BAT-1* DNA fragment. *CRX* mammalian expression vectors *pCAGIG-CRX WT*, *E168d2* or *R90W* and their negative control vector *pCAG-Gfp* (−) were individually transfected into HEK293 cells. A 2-fold dilution series of nuclear protein extract made from each transfection was incubated with 700IRdye-labeled DNA probes, either *BAT-1* or mutated *BAT-1* lacking CRX-binding sites (*BAT-1 Mut AB*) (sequence below EMSA) [7]. The resulting protein/DNA complexes were resolved on 5% non-denaturing PAGE gels and imaged on the LI-COR Odyssey system. Novel bands corresponding to protein/DNA complexes containing full-length CRXs (FULL, either WT or R90W), truncated CRX (E168d2) and non-specific (N.S.) protein(s), as well as free probe (F.P.) are indicated. **B**. Western blot for the amount of CRX protein (antibody 119b-1) present in each nuclear extract. To compare binding activity, CRX protein levels from different nuclear extracts were normalized to the WT level and equal ratios were used for EMSA reactions. **C**. Quantitative chromatin immunoprecipitation assays for promoter occupancy of CRX in P14 *WT* and *E168d2/d2*, *R90W/W* and *−/−* mutant retinas. The indicated target gene promoters were used in qPCR assays on CRX-immunoprecipitated chromatin and the results are presented as enrichment of CRX over IgG control. Like WT protein, both CRX^[E168d2]^ and CRX^[R90W]^ are enriched on the promoters analyzed. **D–E**. Dual-luciferase assays showing combined transactivation activity of NRL, CRX, CRX^[E168d2]^ and CRX^[R90W]^ in transfected HEK293 cells on two promoter-luciferase reporters, *Rhodopsin* (*Rho*, *BR-130* [47]) (**D**) or *Crx* (*0.5K* mouse *Crx*) (**E**). Comparing to *pcDNA3.1/HisC* control, for *Rho*: all test plasmid combinations were significantly different; for *0.5K Crx*: only *pCAG-E168d2* and *pCAG-R90W* were not significantly different. Significant differences of post hoc comparisons are indicated by bracketed ‘*’ (FDR p<0.09; comparisons were made to the left most bracket; Error bars: STDEV).

To determine how mutant forms of CRX protein affect target gene transcription, we assessed their ability to bind to DNA and transactivate transcription. First, electrophoretic mobility shift assays (EMSA) were used to measure DNA binding activity of CRX WT, CRX^[E168d2]^ and CRX^[R90W]^ protein expressed in HEK293 cells on the rhodopsin promoter target site *BAT-1* [1] (Figure 10A). To compare relative binding affinity, the amount of CRX in each nuclear extract was quantified using Western blots and equalized between transfections (Figure 10B). EMSA was then performed on a 2-fold dilution series of nuclear extracts of each CRX protein. Following incubation with *BAT-1* probe, WT CRX extract produced a single species of specific band shift (marked as ‘WT’) with a concentration-dependent intensity. This shifted band represented specific binding of the indicated CRX protein to *BAT-1* CRX sites, as it is absent in the lane receiving the GFP control extract and when the probe contains mutated CRX binding sites (*BAT-1 Mut AB*). CRX^[E168d2]^ nuclear extract also produced a specific band shift (marked ‘E168d2’), which migrated much faster than the full-length CRX band as expected for a truncated protein. The intensity of the E168d2 band was comparable to the WT full-length band at each corresponding concentration, suggesting that CRX^[E168d2]^ binds target sites with similar efficiency as WT CRX, providing a basis for competition binding to common targets. In contrast, CRX^[R90W]^ nuclear extract produced a faint band with the same mobility as WT (Figure 10A), but significantly reduced intensity (∼69% lower than WT). Reduced but not abolished DNA binding activity was also reported for bacterially expressed CRX homeodomain peptides carrying the *R90W* mutation [47]. These results support the hypothesis that CRX^[E168d2]^ protein maintains normal DNA binding ability, while CRX^[R90W]^ protein has reduced DNA binding ability.

To determine if *in vitro* DNA binding activity of each mutant reflected ability to associate with target chromatin *in vivo*, the association of WT CRX, CRX^[E168d2]^ and CRX^[R90W]^ protein with target gene promoter regions was examined using chromatin immunoprecipitation (ChIP) assays. ChIP was performed on P10 mouse retinas of *WT*, *E168d2/d2*, *R90W/W* and *−/−* mice using the CRX 119b-1 antibody [7]. As expected, enrichment of CRX^[E168d2]^ protein was detected on the promoter of genes expressed in rods (*Rho*, *Gnat1*), cones (*Arr3*, *Opn1mw*, *Opn1sw*) and both rods/cones (*Crx*, *Rbp3* (Accession: AJ294749.1)) (Figure 10C, red bars). Despite reduced DNA binding activity in vitro, CRX^[R90W]^ protein was found on the promoter of all candidate genes tested (Figure 10C, green bars). The mechanism by which CRX^[R90W]^, which has reduced DNA-binding ability, is recruited to target gene chromatin *in vivo* remains to be determined. However, these results are consistent with *R90W*'s hypomorphic effect on target gene expression in the retina (Figure 8, Figure 9).

The ability of CRX^[E168d2]^ and CRX^[R90W]^ proteins to transactivate target promoters, either alone or in combination with WT CRX, was assessed by dual-luciferase reporter assays in transiently transfected HEK293 cells. Consistent with a previous report [47], WT CRX was able to cooperate with NRL to activate a *Rhodopsin* promoter-driven luciferase reporter, *BR130* (Figure 10D). However, CRX^[E168d2]^ failed to increase transactivation above NRL alone, suggesting that CRX^[E168d2]^ was unable to form functional interactions with transcription co-activators despite its normal DNA binding ability. In contrast, CRX^[R90W]^ weakly promoted NRL-mediated transactivation, consistent with CRX^[R90W]^'s weak ability to bind target DNA (Figure 10A) and interact with NRL [11] *in vitro* to promote low levels of gene expression in the retinas of homozygous *R90W* mice (Figure 8). To test the effect of mutant protein on WT CRX function, *E168d2* and *R90W* expression vectors were each co-transfected at increasing concentrations with WT CRX. CRX^[E168d2]^ protein significantly impaired WT CRX function when the ratio of *E168d2:WT* vector reached 2∶1 or higher, suggesting CRX^[E168d2]^ actively interfered with WT CRX via an antimorphic mechanism, consistent with the dose-dependent toxicity observed in *E168d2/+* and *E168d2neo/+* mice. In contrast, at the same *mutant:WT* vector ratios, CRX^[R90W]^ protein did not disrupt WT protein function, consistent with the hypomorphic effect of *R90W* in mice.

The *Crx* promoter is another known CRX direct target. It contains two CRX consensus binding sites within a 500-bp upstream region that is required for CRX auto-activation [57]. However, unlike *Rhodopsin*, which is downregulated, *Crx* was overexpressed in *E168d2* mice (Figure 2). To determine if *Crx* overexpression resulted from the direct action of CRX^[E168d2]^ protein on the *Crx* promoter, dual-luciferase reporter assays using the 0.5K *Crx* promoter were performed (Figure 10E). As expected, WT CRX protein transactivated this in a concentration-dependent manner (Figure 10E), while CRX^[E168d2]^ and CRX^[R90W]^ at the highest concentration did not transactivate. When both WT and mutant proteins were present, CRX^[E168d2]^ interfered with the transactivation activity of WT CRX, even at a 1∶2 mutant:WT vector ratio. CRX^[R90W]^ protein also reduced WT CRX transactivation activity, though less strongly, at the 1∶1 and 2∶1 mutant:WT vector ratios. These results suggest that both CRX^[E168d2]^ and CRX^[R90W]^ proteins 1) are less effective than WT CRX at activating target promoters, and 2) interfere with WT CRX autoactivation.

Taken together, functional analyses of CRX^[E168d2]^ and CRX^[R90W]^ proteins revealed that they affected target gene transcription via distinct mechanisms. While CRX^[E168d2]^ bind DNA equally well as WT CRX, it fails to activate transcription and interferes with WT CRX function, resulting in a dose-dependent antimorphic effect. In contrast, CRX^[R90W]^ has reduced ability to bind target DNA and regulate transcription, qualifying CRX^[R90W]^ as a hypomorphic protein.

## Discussion

### *E168d2* and *R90W* mouse lines accurately model the corresponding human diseases

#### *E168d2* mice model dominant ‘LCA’ or severe early-onset ‘CoRD’

While all homozygous *K-IN* mice assessed in this paper demonstrated ‘LCA’-like phenotypes, only *E168d2/+* mice presented with dominantly inherited retinopathy characteristic of ‘LCA’ or severe early-onset ‘CoRD’. Rods and cones of *E168d2/+* mice were strongly functionally impaired from 1 mo, exhibited abnormal nuclear and OS morphology and degenerated rapidly within the first 6 mo of life (Summarized in Table 2). Importantly, the cone deficits were more severe and occurred earlier than rod deficits. Cone nuclei were mislocalized to the inner ONL at P14 and 1 mo (Figure 5E&F) and the number of cones was decreased (32.3% of WT) in *E168d2/+* mice at 1 mo (Figure 6B). In addition, the cone opsin gradient [56] was highly disrupted in *E168d2/+* and *E168d2neo/+* mice (Figure 6M–P). Misregulation of this gradient could arise either from inability of mutant CRX proteins to properly interact with co-factors to cooperatively regulate opsin expression, or as a result of impaired cone subtype specification. The cone opsin gradient is regulated by multiple transcription factors including RXRγ (Accession: AAH13709.1) [58] and TRβ2 (Accession: NP_033406.1) [59]. Expression of *Rxrγ* and *Trβ2* in *E168d2/+* or *E168d2neo*/+ retinas at P21 was normal as measured by qRT-PCR (Table S6), although *Rxrγ* expression was elevated at P10 (Table S5). While these data suggest terminal differentiation of cones in *E168d2/+* retina may have been compromised, it is unclear if cone deficits were due to impaired cell fate specification, survival, or both. The accurate quantification of cone number was not possible in *E168d2/+* retina at P14 or earlier due to reduced expression of cone markers including cone opsins. Future experiments tracing the cone lineage in developing *E168d2/+* retinas are needed to distinguish these possibilities.

**Table 2:** Summary of retinal phenotypes of *Crx E168d2*, *R90W* and *KO* mice.

	Function (ERG)		OS Length	Nuclear position	Opsin Gradient	Photoreceptor degeneration timecourse		Gene expression		Disease model
Genotype	Rod	Cone	Rod	Cone	Cone	Rod	Cone	P10	P21	
***WT***	++++	++++	++	OONL	+++	-	-	+++	+++	
***E168d2/*** **+**	+	+	+	MONL	-	1–6 mo	1 mo	+	+	LCA
***E168d2neo/*** **+**	+++	++	++	IONL	+	-	1 mo-1 yr	+	++	CoRD
***R90W/*** **+**	++++	++++	++	OONL	++	-	-	++	+++	Mild CoRD
**+*/*−**	++++	++++	++	OONL	ND*	-	-	++	+++	
***E168d2/d2***	-	-	-	-	-	1–3 mo	1–3 mo	-	-	LCA
***R90W/W***	-	-	-	-	-	1–3 mo	1–3 mo	-	-	LCA
**−*/*−**	-	-	-	-	-	1–3 mo	1–3 mo	-	-	LCA

‘+’ qualitative graded levels; ‘−’ null;

* ND: Not determined.

In summary, the *E168d2/+* mouse phenotype closely matched the clinical features of patients diagnosed with LCA who carry the *E168d2* mutation. These patients showed severe vision loss detectable within the first few months of life, including markedly reduced ERG responses [24] [31] [41]. Several pieces of evidence suggest that the *E168d2/+* mouse also models human disease associated with other mutations within the class of frameshift truncation mutations. First, several other frameshift and deletion mutations in human *CRX* caused similar clinical phenotypes [22]–[33] [36]–[40]. Second, *in vitro* functional analyses showed that these mutations resulted in defects in target gene transactivation similar to those we see with *E168d2* [12] [47]. Third, the recently-identified feline model *Rdy*, which also carries a frameshift truncation mutation in *Crx* [60], has severely reduced visual function and progressive photoreceptor degeneration that closely matches the *E168d2/+* phenotype [61]. *Rdy* cats carry a spontaneous single base-pair deletion *n.546delC* producing a truncated CRX protein just 14 amino acids longer than the CRX^[E168d2]^ protein [60]. Thus, a common pathogenic mechanism is likely responsible for these similar phenotypes in different mammalian species, for which the *E168d2* mouse serves as an appropriate small-animal model.

#### *E168d2neo* reveals that expression levels of mutant CRX correlate with disease severity

*CRX*-associated dominant diseases vary in age of onset and severity, even with similar mutations [22] [23]. The factors responsible for these phenotype variations have not yet been identified, but comparing findings from *E168d2* and *E168d2neo* mice revealed one possible mechanism. The *E168d2neo* mouse, which had reduced expression of CRX^[E168d2]^, showed a less severe phenotype than *E168d2*, resembling later-onset dominant ‘CoRD’. Characterization of the low expression subline *E168d2neo/+* (which expressed 30% less mutant protein) in parallel with *E168d2/+* revealed that mutant allele expression level significantly impacted phenotype severity. In young *E168d2neo/+* mice, the rod phenotype was almost fully rescued and the cone phenotype was significantly improved by all measures performed, including rod and cone morphology (Figures 4–6), function (Figure 7, Table 1), survival (Figure S2) and gene expression (Figure 9, Table S5, Table S6). While older *E168d2neo/+* mice did show impaired cone function and degeneration, disease progression was much slower compared to *E168d2/+*. Thus, while the *E168d2* mouse represented the most accurate disease model for the human *CRX^E168d2^* phenotype, the *E168d2neo/+* mouse modeled less severe forms of ‘CoRD’. The expression level-dependent phenotypes of the *E168d2* mouse lines have several implications: 1) These findings support an antimorphic activity for CRX^[E168d2]^ protein; 2) In human patients carrying similar *CRX* mutations, features intrinsic to the mutation allele and/or genetic background may affect *CRX* expression, which could impact disease severity; 3) Consequently, therapy directed at shifting the ratio of WT to mutant CRX protein might be effective at improving vision in patients.

#### Overexpression of CRX^[E168d2]^ protein in *E168d2* mice is caused by unknown mechanism

*E168d2* mice overexpressed both *Crx* mRNA and protein in an allele specific manner (Figure 2H–J), indicating *Crx* misregulation occurred at the RNA level, either in the synthesis or degradation of the mRNA transcript. Transient transfection assays (Figure 10E) showed that while WT CRX was able to transactivate its own promoter, CRX^[E168d2]^ had lost transactivation activity and interfered with WT CRX autoregulation. Thus, overexpression of the *E168d2* allele in vivo was unlikely due to the direct action of CRX^[E168d2]^ protein on the *Crx* promoter. Other possible mechanisms for *E168d2* allele-specific overexpression *in vivo* include suppression of a negative feedback regulation, changes in transcription efficiency of the *E168d2* allele, or stability of the *E168d2* transcript. Expression of a known regulator of *Crx* expression, *Otx2* (Accession: NM_144841.3), was altered in *E168d2* mice. *Otx2* is required for induction of *Crx* expression during development [49] [62] but is normally turned off in differentiated photoreceptors when CRX expression reaches high levels. *Otx2* was upregulated at P21 in *E168d2*/+ retinas (Table S5, Table S6), suggesting that the feedback network was affected in the mutants. A more comprehensive investigation of the network regulating *Crx's* transcription is required. Alternatively, since the level of RNA made by the mutant allele was much higher than that of the *WT* allele in *E168d2/+* retinas, it is plausible that changes in *Crx E168d2* mRNA stability may be involved. More importantly, since this mutant allele-specific overexpression was shared by anther frameshift mutation, *I138^fs48^* in *Drosophila* [12] [46], the underlying molecular mechanism could be conserved for this type of CRX mutation.

#### *R90W* mice model mild late-onset dominant ‘CoRD’

While homozygous *R90W* mice displayed a ‘LCA-like’ phenotype, heterozygous *R90W/+* mice didn't exhibit changes in photoreceptor morphology, gene expression or degeneration at the early ages tested (Table 2). However, minor changes in cone function were detectable at 6 mo (Figure 7I), suggesting *R90W/+* has a mild dominant phenotype. The *R90W/+* phenotype closely resembled the clinical features of a pedigree where the equivalent human *R90W* mutation was identified [45]: A proband homozygous for *R90W* had almost complete loss of vision and was diagnosed with autosomal recessive LCA. Her parents, each of whom carried one *R90W* allele, had mild cone functional defects and reduced color discrimination ability by middle age (40's). It is unknown if the cone functional defects reported for the human carriers are related to misregulation of cone gene expression or cone degeneration. Several other substitution mutations were similarly associated with mild late-onset dominant disease in humans [12] [22] [23] [33] [36] [42] [45]. Overall, reduced numbers of rods or cones were not observed in *R90W/+* mice up to one year of age, but disease may appear at later ages. The mild dominant phenotype of *R90W/+*, not observed in *+/−* mice, as well as the *CRX* promoter-driven luciferase assay results (Figure 10E) suggest CRX^[R90W]^ possesses some minor disruptive effects on WT CRX function. Overall, however, unlike *E168d2* and *E168d2neo*, there were no major phenotypic differences observed between *R90W* and *R90Wneo*, consistent with CRX^[R90W]^ being predominantly a hypomorphic protein. It is unknown how genetic background or environmental interactions contribute to the substitution mutation disease phenotype. The *R90W* mouse line provides a valid small animal model for investigating this subset of milder *CRX*-related diseases.

### CRX^[E168d2]^ and CRX^[R90W]^ protein cause disease through distinct mechanisms

**Figure 11:**
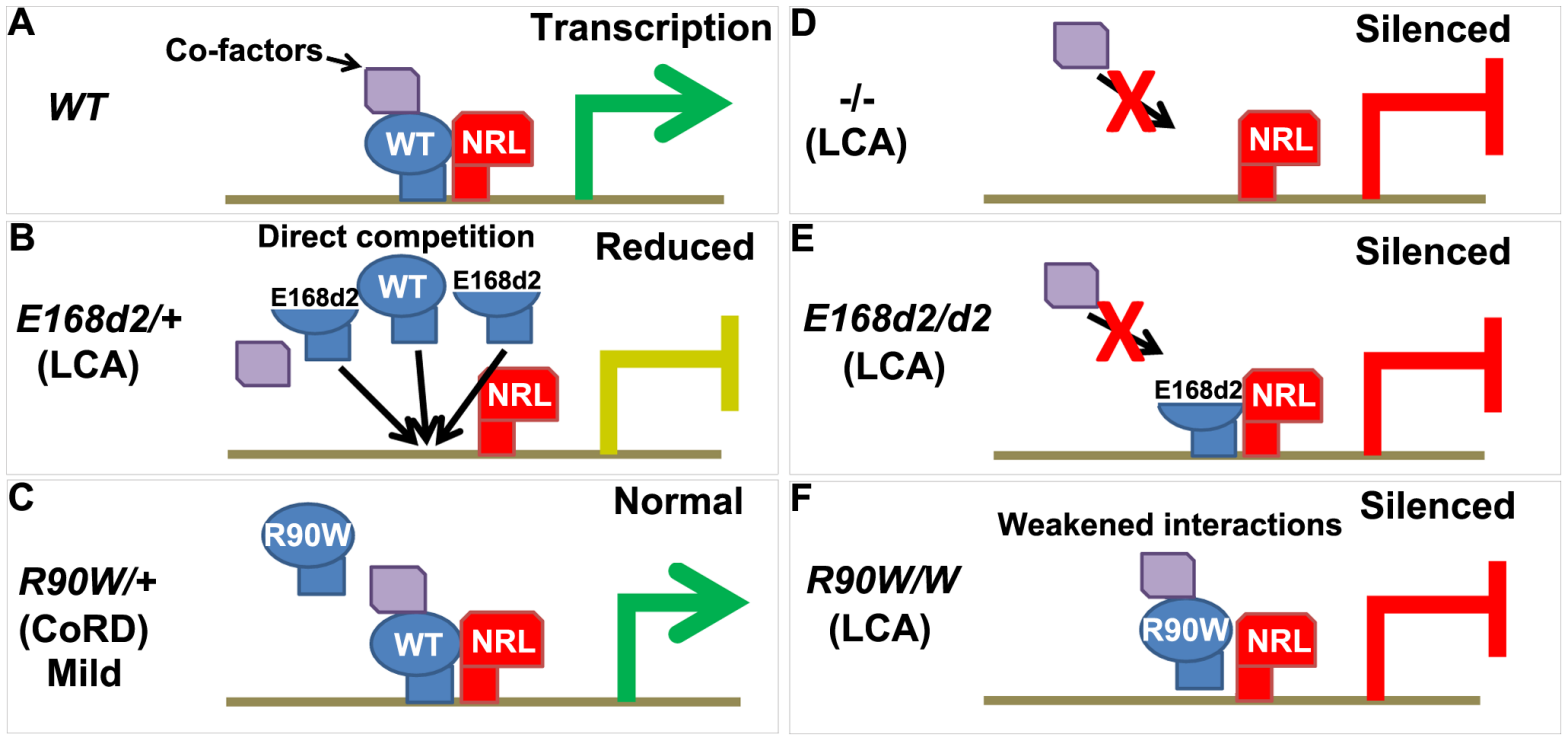
Models for CRX^[E168d2]^ and CRX^[R90W]^ mechanisms of pathogenesis in *K-IN* mice. **A**. In *WT* mice, CRX binds to DNA, recruits and synergizes with co-factors including NRL and chromatin modulators [7] to promote target gene transcription. **B**. In *E168d2/+* mice, the antimorphic CRX^[E168d2]^ protein directly competes with CRX WT to act on target gene promoters. Since CRX^[E168d2]^ protein is overexpressed compared to WT, its antimorphic effect is amplified and results in severe reductions in target gene transcription and retinopathy resembling LCA. **C**. In *R90W/+* mice, CRX^[R90W]^ protein does not impair the function of CRX WT and transcription in adult mice is largely normal. *R90W/+* mice have only a mild retinal phenotype similar to a late-onset CoRD. **D**. In −/− mice, the loss of CRX leads to the failure to recruit co-factors to target gene promoters and expression is silenced [7] [19]. **E**. In *E168d2/d2* mice, CRX^[E168d2]^ protein binds to the promoters of target genes, which interferes with the activity of other transcription factors, resulting in early-onset LCA with a faster course of retinal degeneration than in −/− mice. **F**. In *R90W/W* mice, CRX^[R90W]^ protein still associates with target gene promoters despite having reduced DNA binding in vitro. However, this confers only modest gains in target gene transcription over −/−, insufficient to establish normal retinal function. Thus, CRX^[R90W]^ protein is a functionally impaired protein but retains some residual transactivation activity. *R90W/W* mice most closely model LCA.

Several pieces of evidence support that CRX^[E168d2]^ and CRX^[R90W]^ protein cause disease via different mechanisms, as illustrated in Figure 11. CRX^[E168d2]^ protein bound to DNA, interfered with the function of CRX WT and impaired the expression of CRX target genes, classifying it as an antimorphic protein with dominant-negative activity (Figure 11B). All of our results suggest that CRX^[E168d2]^'s activity was largely restricted to CRX target genes. Of the 82 uniquely downregulated genes identified in homozygous *E168d2neo* mice, most (76.8%) also exhibited direct CRX binding. The average fold change of these distinct genes was less dramatic than genes shared between *E168d2* and −/−, suggesting they were likely to be similarly affected in −/− but failed to pass the significance threshold. Many shared genes including: *Rho*, *Arr3*, *Ramp3*, *Drd4*, *Cpm*, and *Pde6c* were more strongly downregulated in homozygous *E168d2neo* than −/− mice (Figure 8D). This suggests CRX^[E168d2]^ protein had an antimorphic effect on the expression of these genes even in the complete absence of WT CRX, possibly by interfering with other co-factors like the homeodomain transcription factor OTX2. Supporting this hypothesis, removal of one allele of *Otx2* from the −/− mouse produced a severe phenotype similar to homozygous *E168d2* mice [63]. Since OTX2 and CRX have overlapping spatial and temporal roles in retinal development and share DNA binding domain homology, it is possible that CRX^[E168d2]^ interfered with OTX2 activity, resulting in a stronger phenotype than −/−. This antimorphic effect is unlikely to involve interference with NRL function, since NRL expression was comparable in all homozygous models (Table S5), CRX^[E168d2]^ did not interfere with NRL transactivation (Figure 10D) and a similar truncation mutation in bovine CRX C160 (1–160) maintained interaction with NRL [11]. qRT-PCR analysis of CRX target gene expression showed downregulation that correlated with mutant CRX expression level (Figure 9), supporting the conclusion that CRX^[E168d2]^ is an antimorphic mutant protein with dominant negative activity. The *E168d2* mouse model thus demonstrates the effects of an antimorphic truncated CRX protein associated with human disease.

The CRX^[R90W]^ protein had reduced DNA binding and weakly promoted transcription *in vitro*, classifying CRX^[R90W]^ as a hypomorphic protein (Figure 11C&F). Although binding of CRX^[R90W]^ to the BAT-1 oligo *in vitro* was reduced (Figure 10A), CRX^[R90W]^ associated with CRX target DNA *in vivo* (Figure 10C), suggesting co-factors may anchor CRX^[R90W]^ to target DNA. CRX^[R90W]^ weakly promoted NRL-mediated transactivation of the *Rho* promoter *in vitro* (Figure 10D), consistent with early findings that CRX^[R90W]^ protein reduced the physical interaction with NRL [11]. Thus, even though CRX^[R90W]^ was associated with target promoters *in vivo*, it may have lost specific interactions with co-factors, therefore reducing its function. Indeed, despite being present on target promoters, CRX^[R90W]^ only weakly promoted target gene expression *in vivo*, as shown by reduced expression of many CRX target genes in homozygous *R90W* retinas as detected by microarray (Figure 8A–E) and qRT-PCR (Figure 8J–M). However, target gene expression in *R90W* retinas was less reduced compared to −/− retinas (Figure 8D, J–M), suggesting CRX^[R90W]^ possessed some residual transcriptional activation activity. In Drosophila, human *R90W* was able to partially rescue the otd^uvi^ phenotype, consistent with a hypomorphic mechanism [46]. Taken together, our results show that CRX^[R90W]^ is a predominantly hypomorphic mutant CRX protein, representative of substitution mutations associated with mild forms of CRX disease.

### Mechanistically distinct mutations underlie *CRX*-associated disease

The molecular functions of several CRX mutations associated with human retinopathy have been investigated *in vitro* [12] [45] [47] and *in vivo* in Drosophila [46]. Such studies indicate that mutant CRX proteins have distinct molecular functions, which could in part explain the variation in *CRX*-disease phenotypes. The distinct phenotypes of mice carrying *E168d2*, an antimorphic frameshift mutation, and *R90W*, a hypomorphic substitution mutation, further expand our understanding of the impact of mutation type on disease pathology and closely match the functions and associated phenotypes of other similar type mutations. This suggests that *E168d2* and *R90W K-IN* mice are representative animal models for two larger groups of disease causing mutations, increasing their utility as research tools for studying pathology and developing therapies. There are likely additional mechanisms of *CRX*-associated disease yet to be modeled *in vivo*, such as substitution mutations that do not affect DNA-binding but are nonetheless associated with dominant disease [12] [45] [47]. Collectively, these studies demonstrate the diversity of molecular defects mediating CRX-associated disease and highlight the value of having multiple small-animal models to understand them.

### *Crx E168d2*, *E168d2neo* and *R90W* provide distinct models for therapy development

Currently, there are no treatment strategies for *CRX*-associated diseases. Since CRX influences many cellular processes, designing targeted therapy is exceptionally difficult. The availability of phenotypically and mechanistically distinct models for *CRX*-associated disease will greatly improve our ability to develop novel therapies. *E168d2*, *E168d2neo* and *R90W* present unique mechanistic challenges for therapy to address. Stem cell based therapies have previously been shown to restore function in the −/− mouse [64]. Like −/− mice, *E168d2*/+, *E168d2/d2* and *R90W/W* mice all have highly abnormal photoreceptor morphology and undergo rapid degeneration, which may restrict the time course and effectiveness of treatment. The improved phenotype of *E168d2neo*/+ mice, compared to *E168d2/+*, provides evidence that gene replacement strategies that shift the ratio of WT to mutant CRX could be effective at improving vision and promoting rod and cone survival in cases were a mutant protein is toxic and/or overexpressed. Previous studies have shown this strategy to be effective in treating a dominant-negative adRP RHO animal model [65] [66]. Lastly, the similarity of the *E168d2*/+ mouse and the *Rdy/+* cat provide excellently matched small and large animal models. Therapies that are proven to be effective in the *E168d2/+* mouse can immediately be tested in the *Rdy/+* cat, which improves our ability to develop translational therapies.

In summary, *Crx E168d2* and *R90W* are mechanistically distinct mouse models for *CRX*-associated disease, demonstrating how different classes of *CRX* mutations yield drastically different retinal phenotypes. *E168d2* and *R90W* accurately recapitulate human diseases caused by distinct classes of human mutations and have greatly improved our understanding of disease pathobiology. The availability of these stratified mouse models for *CRX*-associated disease is an invaluable resource for developing effective mechanism based therapies.

## Materials and Methods

### Ethics statement

All procedures involving mice were approved by the Animal Studies Committee of Washington University in St. Louis, and performed under Protocols # 20090359 and 20120246 (to SC). Experiments were carried out in strict accordance with recommendations in the Guide for the Care and Use of Laboratory Animals of the National Institutes of Health (Bethesda, MD), the Washington University Policy on the Use of Animals in Research; and the Guidelines for the Use of Animals in Visual Research of the Association for Research in Ophthalmology and Visual Science (http://www.arvo.org/animals/). Every effort was made to minimize the animals' suffering, anxiety, and discomfort.

### Mice

Mice were housed in a barrier facility operated and maintained by the Division of Comparative Medicine of Washington University School of Medicine. All mice used for experiments were backcrossed to *C57BL6/J* mice obtained from Jackson Laboratories (Bar Harbor, ME, Stock number 000664) for at least 5 generations. Knock-IN of *E168d2neo* and *R90Wneo* were generated by the Mouse Genetics Core, Department of Ophthalmology and Visual Sciences, Washington University (Saint Louis, MO). *E168d2neo* and *R90Wneo* constructs were transfected into *129Sv/J* SCC#10 (ATCC SCRC-1020) embryonic stem cells and Knock-IN was achieved by homologous recombination into the endogenous *mCrx* locus and selected by *neomycin*. The targeted ES cells were injected into *C57BL6/J* blastocysts to form chimeric *Knock-IN E168d2neo* and *R90Wneo* mice. Germline transmission of *E168d2neo* and *R90Wneo* was identified by PCR genotyping and Sanger sequencing of genomic DNA from F1 mice (Figure 1, Figure S1, Table S1). *Crx^−/−^* mice were provided by Dr. Constance Cepko, Harvard University (Boston, MA).

### PCR genotyping

Genomic DNA was prepared from mouse tail tissue using the Gentra Puregene Tissue Kit (Qiagen). PCR amplification was performed using Jumpstart RedTaq (Sigma-Aldrich). Primer sets (Table S1) are as follows: For all mice: *neo* (Neo-F/R) and Crx (Total Crx-F/R); for *E168d2* colony: *WT Crx* allele (E168d2 WT-F, E168d2-R), *E168d2* allele (E168d2 Mut-F, E168d2-R); for *R90W* colony: *WT Crx* allele (R90W WT-R, R90W-R), *R90W* allele (R90W Mut-F, R90W-R).

### Sanger sequencing of genomic DNA

Genomic DNA was prepared from mouse tail tissue using the Gentra Puregene Tissue Kit (Qiagen). *mCrx* DNA was amplified by PCR using the Genomic *mCrx* Int/Ex4-F/R primer pair (Table S1). Sanger sequencing was performed by the Protein and Nucleic Acid Chemistry Laboratory (Washington University, Saint Louis, MO) using the Sequencing primers *E168* and *R90W* (Table S1) and Big Dye V3.1 (Advanced Biotechnologies).

### Electroretinogram

At least 5 mice of each genotype were tested for ERG at 1 mo, 3 mo, or 6 mo of age. Bilateral flash ERG measurements were performed using a UTAS-E3000 Visual Electrodiagnostic System running EM for Windows (LKC Technologies, Inc., Gaithersburg, MD) and recordings from the higher amplitude eye were used for analysis. Mice were dark-adapted overnight, then anesthetized with 80 mg/kg ketamine and 15 mg/kg xylazine under dim red illumination for electrode placement and testing. Body temperature was maintained at 37±0.5°C with a heating pad controlled by a rectal temperature probe (FHC Inc., Bowdoin, ME). The mouse's head was positioned just inside the opening of the Ganzfeld dome and pupils were dilated with 1.0% atropine sulfate (Bausch & Lomb, Tampa, FL). The recording electrode was a platinum loop 2.0 mm in diameter, positioned in a drop of 1.25% hydroxypropyl methylcellulose (GONAK; Akorn Inc., Buffalo Grove, IL) on the corneal surface of each eye. The reference needle electrode was inserted under the skin at the vertex of the skull. The ground electrode was inserted under the skin of the mouse's back or tail. The stimulus (trial) consisted of a brief, full-field flash (10 µs) either in darkness, or in the presence of dim (29.2 cd/mm) background illumination after 10 minutes adaptation time to the background light. The initiation of the flash was taken as time zero. The response was recorded over 250 ms plus 25 ms of pre-trial baseline. Responses from several trials were averaged. For complete test parameters see Table S7. The log light intensity (log [^cd*s^/_m_^2^]) was calculated based on the manufacturer's calibrations. The mean amplitudes (in microvolts) of the averaged dark-adapted A and B-waves and light-adapted B-waves were measured and quantified for comparison. The between-group differences in peak amplitude were determined by testing genotype*flash intensity interactions (p<0.05, n≥5) at each age were compared using two-way ANOVA for repeated measurement data to account for potential correlations among readings from the same mice. If the overall genotype*flash intensity interaction was significant, post-hoc multiple comparisons for differences between each genotype and the control group at each light intensity level were performed. All the tests were two-tailed, significance: p<0.05. The statistical analysis was performed using SAS 9.3 (SAS Institutes, Cary, NC). p-values were adjusted for multiple comparisons by a permutation test using the default parameters provided in the LSMestimate statement in Proc Mixed. Average percent reductions for each wave form were calculated by normalizing the peak amplitude of the mutant to *WT* and results were averaged for the flashes listed in Table 1; ±STDEV.

### Immunohistochemistry and microscopy

For retinal sections: eyes were enucleated by removing the cornea and lens and fixed in 4% paraformaldehyde for 24 hrs at 4°C. A small corneal tag on the superior portion of the eye was used for orientation. Eyes were embedded in paraffin and 5 µM sagittal retinal sections were cut using a Leica RM 2255 microtome as previously described [67]. Hemotoxylin and eosin immunohistochemistry was performed on sections for histology. Fluorescent antibody immunostaining was performed using as previously described using 1% BSA/0.1% Triton X in 1× PBS for blocking and antigen retrieval for all samples [13] [67].

For whole flat-mounted retinas: eyes were enucleated by removing the cornea and lens and fixed in 4% paraformaldehyde for 1 hr at 4°C. Retinas were then dissected from the eye cup and 4 evenly spaced relief lines were cut (Figure 6A). A scleral tag was left on the superior retina for orientation. Retinas were mounted on poly-D lysine coated slides (Thermo Scientific), blocked with 1% BSA/0.1% Triton X in 1× PBS and immunostained as previous.

Primary antibodies and dilutions used as follows: Mouse monoclonal anti-CRX M02 (1∶200, Abova), rabbit anti-CRX 261 (1∶200), rabbit anti-cone arrestin (CARR) (1∶1000, Millipore), Rabbit anti-Opsin Red/Green (MOP) (1∶1000, Millipore), Goat anti-OPN1SW (N-20) (SOP) (1∶500, Santa Cruz), Mouse anti-Rhodopsin RET-P1 (RHO)(1∶400, Chemicon), Peanut Agglutanin conjugated to Rhodamine (PNA)(1∶500, Vector Labs). Secondary Antibodies (1∶400): Goat anti-rabbit or mouse IgG antibodies coupled to Alexa Fluor A488, Rhodamine 568 or Cy2 647 (Molecular Probes) and Chicken anti-goat IgG (Molecular Probes). All slides were counterstained with hard set DAPI (Vectashield), except when using Cy2 secondary, which were counterstained with Slow Fade Gold DAPI (Invitrogen). All brightfield and fluorescent imaging was performed using an Olympus BX51 microscope and Spot RT3 Cooled Color Digital camera (Diagnostic instruments inc.).

TUNEL analysis was performed using the Apoptag Fluorescein in situ Apoptosis Detection Kit (Millipore) per kit instructions. TUNEL+ cells were counted in retinal sagittal sections of P21 and P35 mice. Significant differences from *WT* control (p<0.05) were determined by the Kruskal-Wallis rank order test, which was used to protect against departures from the normal distribution assumption.

### Morphometric ONL and cone analyses

For ONL morphometry, 20× retinal composites of hematoxylin and eosin (H&E) stained sagittal sections were analyzed using Image J software (http://rsb.info.nih.gov/ij/). The distance from the Optic Nerve (ON) was determined by drawing a curved line along the outer limiting membrane. The ONL thickness was measured at 100 µM, 500 µM, 1000 µM, and 1500 µM from the ON and 200 µM from the peripheral edge on both the superior and inferior retina. Results are presented by ‘spider graph’. The between-group differences in ONL thickness were determined by testing overall genotype*distance interactions (p<0.05, n≥3) at each age were tested using two-way ANOVA for repeated measurement data, followed by a post-hoc test to adjust p-value for multiple comparisons between each genotype and the *WT* control group at each distance using SAS 9.3 (SAS Institutes, Cary, NC), as above.

Cone nuclear localization was determined by immunostaining retinal sections with CARR. The ONL was divided into 3 equally sized zones (OONL, MONL, IONL; Figure 5A) on 20× retinal composite images using Image J software (http://rsb.info.nih.gov/ij/) and the cone nuclei within in each zone from three sections for each mouse were counted. Significant differences from *WT* for each zone were determined by Kruskal-Wallis rank order test (p<0.05, n≥3)

For cone density and opsin expression assessment, 10 images at 40× magnification of whole flat-mounted retinas were taken in the zones specified in Figure 6A. All peripheral images were taken ∼400 µM from the edge of the retina and the central image was taken ∼250 µM from the ON along the lateral axis. Cones were counted within a 200×200 µM square grid for each image using Image J software and the density of cones/(mm^2^*1000) was calculated. The between-group differences in cone density were determined by testing overall genotype*retinal region interactions (p<0.05, n≥3) at each age were tested using two-way ANOVA for repeated measurement data, followed by a post-hoc test to adjust p-value for multiple comparisons between each genotype and the *WT* control group in each retinal region using SAS 9.3 (SAS Institutes, Cary, NC), as above. For regional cone opsin expression analysis (Figure 6E–P), differences in the fraction of cones expressing SOP, MOP, SOP/MOP or no opsin was tested in each region using a Kruskal-Wallis rank order test (p<0.05).

### Transmission electron microscopy (TEM)

For TEM studies, eyes were enucleated by removing the cornea and lens and fixed in 2% paraformaldehyde/3% gluteraldehyde in 0.1 M phosphate buffer (pH 7.35) for 24 hrs, post-fixed in 1% osmium tetroxide for 1 hr and stained *en bloc* with 1% uranyl acetate in 0.1 M acetate buffer for 1 hr. Blocks were then dehydrated in a graded series of acetones and embedded in Araldite 6005/EMbed 812 resin (Electron Microscopy Sciences). Semi-thin sections (0.5–1 µm) were cut through the entire retina at the level of the optic nerve and stained with toluidine blue, post-stained with uranyl acetate and lead citrate, viewed on a Hitachi H7500 electron microscope and documented in digital images. Three retinas for each genotype were sampled at P21 at 800–1200 µM from the optic nerve. ≥10 images of four key features were collected by random sampling: OS-RPE (10000×), OS-IS (12000×), ONL (5000×), OPL (10000×). Images were analyzed in a blinded manner using Image J software.

The nuclear percent area of heterochromatin was measured using Image J software in a randomized blinded analysis. For each genotype, 10 5000× images of the ONL were taken for three mouse retinas. For each image, 10 rod nuclei were randomly selected for analysis. The rod nucleus was outlined using the segmented polygon tool, electron dense regions of the nuclei associated with heterochromatin were thresholded and the percentage of the area above the threshold was measured. Thresholding was variably adjusted to accommodate for differences in brightness and contrast. The between-group differences were compared using one-way ANOVA for repeated measurement data, to account for potential correlations among photos from the same mouse. All the tests were two-tailed, significance: p<0.05 (n = 3). The statistical analysis was performed using SAS 9.3 (SAS Institutes, Cary, NC). The overall test for genotype difference was statistically significant (p = 0.02), therefore *E168d2*/+ and *E168d2neo*/+ were compared to *WT* (Figure S2).

### Transient transfection assays

HEK293 cells (ATCC CRL-11268) were cultured on 60 mm plates in Dulbucco's minimum essential media (DMEM) with 10% fetal bovine serum and Penicillin/Streptomycin. Cells in 60% confluence were transfected with *pCAGIG-NRL* and *pCAGIG-hCRX WT*, *E168d2* and *R90W* either alone or in combination using CaCl (0.25 M) and Boric Acid Buffered Saline (1×) pH 6.75 as previously described [13]. Cells were harvested 48 hours post transfection for either RNA (PerfectPure RNA tissue kit, 5Prime), protein (NePER nuclear and cytoplasmic extractions reagents, Thermo Scientific), or Dual-luciferase assays. Dual-luciferase assays were performed as previously described [13]. Significant differences from pcDNA3.1hisc control were determined by Kruskall-Wallis rank order test (p<0.05; n = 3). Post-hoc comparisons (Figure 10 D&E; indicated by brackets) were tested using a less conservative FDR p-value method for multiple comparisons using PROC Multtest of SAS (V9.3). FDR p<0.09 was considered marginally significant.

### Western blot assays

Whole retina protein lysates were prepared by homogenization of four genotype-matched isolated whole retinas from P10 mice and lysis in 1× RIPA buffer (Sigma) for 10 min with protease inhibitors (Aprotinin, Leupeptin, peptistatin, 0.1 mM Phenylmethaneslfonyl fluoride). Nuclear lysates were prepared using NE-PER Nuclear and Cytoplasmic Extraction Reagants (Thermo Scientific) with protease inhibitors. Either 30 µg of whole protein lysate or 5 µg of nuclear protein lysate was boiled for 10 min. Samples were run on a 4–11% SDS-PAGE gel and transferred onto Transblot Turbo nitrocellulose membranes (Bio-Rad) using the Transblot Turbo system (Bio-Rad). Membranes were probed with Rabbit anti-CRX 119b1 (1∶750) and Mouse anti-β-Actin (Sigma)(1∶1000). Goat anti-Mouse IRDye 680LT and Goat anti-Rabbit IRDye 800CW (LI-COR) were used as secondary antibodies. Signal was detected and quantified using the Odyssey Infrared Imager (LI-COR) and associated manufactory software. Kruskal-Wallis rank order test (Proc Npar1way of SAS, V9.3) was used to test for an overall difference among genotypes (p = 0.0002), then each genotype was compared to *WT* control (p<0.05). Post-hoc analyses were performeded using FDR p methods for multiple comparisons using PROC Multtest of SAS (V9.3) (FDR p<0.09) (n≥3).

### qRT-PCR

RNA was extracted from whole retinas of one male and one female mouse at either P10 or P21 for each biological replicate using the PerfectPure RNA tissue kit (5Prime). RNA was quantified using a NanoDrop ND-1000 spectrophotometer (NanoDrop Technologies, Wilmington, DE). cDNA was synthesized from 1 µg of RNA using the Transcriptor First Strand cDNA Synthesis kit (Roche Applied Science). A 10 µl QRT-PCR reaction mixture containing 1× EvaGreen with Low Rox reaction mix (BioRad), 1 µM primer mix, and diluted cDNA was prepared and run on a two-step 40 cycle amplification protocol with melt curve determination on a BioRad CFX thermocycler in triplicate. The Cq's of technical replicates were averaged and the results were analyzed using the Delta Cq method in QBase software (Biogazelle). Primer sets (Table S1) were designed using MacVector software and synthesized by IDT DNA technologies. For *mCrx* allele specific amplification the following primers were used: for *E168d2* and *E168d2neo* mice: WT allele specific- Crx E168d2 WT RTF/R, total- Crx R90W WT-RTF/R; for *R90W* and *R90Wneo* mice: WT allele specific- Crx R90W WT-RTF/R, total Crx E168d2 WT RTF/R (Figure 2J), Relative gene expression was normalized to *Ubb* and *Tuba1b*. Kruskal-Wallis rank order test (Proc Npar1way of SAS, V9.3) was used to test for an overall difference among genotypes (p<0.05; n≥3). Post hoc analyses were adjusted for multiple comparisons using FDR p methods, as above (FDR p≤0.09).

### Microarray

Triplicate RNA samples were prepared from 4 pooled retinas from 1 male and 1 female mouse at P10 for *WT* and homozygous *E168d2neo*, *R90Wneo* and −/− mice. The RNA was fluorescent labeled and hybridized to MouseWG-6 v2.0 Expression Beadchips (Illumina) by Washington University Genome Technology Access Center (GTAC). The raw microarray datasets are available at the NCBI GRO website (http://www.ncbi.nlm.nih.gov/gds, access number: GSE51184). Microarray data were analyzed using significance analysis of microarrays (SAM) following background subtraction and quantile normalization in Illumina Genome Studio platform. Control probes and probes with detection p-value <0.05 across all samples were removed prior to any analysis. Candidate probes with 2.0-fold disregulation at false discovery rate ≤0.05 from each comparison were chosen for further analysis. Cellular processes associated with differentially expressed genes were assigned based on gene ontology provided by Mouse Genome Informatics (http://www.informatics.jax.org/).

### Electrophoretic mobility shift assays (EMSA)

*BAT-1* and *BAT-1* mutated *AB* probes 5′ end-labeled with 700 IRDye were synthesized by Integrated DNA Technologies (IDT). Nuclear protein extracts from HEK293 cells (∼1×10^8^ cells) transfected with *pCAGIG-hCRX*, *pCAGIG-hCRX E168d2*, or *pCAGIG-hCRX R90W* were prepared following NE-PER kit instructions (Thermo Scientific). Nuclear extracts were tested for CRX expression by running on a Western Blot as above (Figure 10B). CRX levels were quantified by normalizing to β-Actin (Sigma) and a 2-fold dilution series of equivalent amounts of CRX WT, CRX^[E168d2]^ and CRX^[R90W]^ protein were used for binding reactions. Binding reactions were performed using the Odyssey Infrared EMSA kit (LI-COR), per kit instructions using 1 µg of nuclear protein extract and 50 nM IRDye labeled oligo. Samples were run on a native 5% polyacrylamide; 0.5× Tris/Borate/Ethylenediaminetetraacetic acid (EDTA) buffered gel and imaged on the Odyssey Infrared Imager (LI-COR).

### Chromatin immunoprecipitation (ChIP) assays

ChIP was performed as previously described [7] [13] [68]. Basically, 6 retinas per sample were dissected and chromatin was cross-linked with 1% formaldehyde in PBS for one minute at room temperature. After cell lysis and chromatin fragmentation by sonication, chromatin fragments were immunoprecipitated with the CRX 119b-1 antibody [7] or normal rabbit IgG (Santa Cruz) bound to Protein A beads (GE Healthcare Life Sciences, Piscataway, NJ). After extensive washing, the immunoprecipitated chromatin was eluted with 50 mM NaHCO_3_ 1% SDS, heated to 67°C to reverse the cross-links, the DNA purified by ethanol precipitation and analyzed by PCR with gene-specific primers (Table S1) (n≥3). Fold enrichment was determined by quantitative ChIP PCR. Critical threshold (Ct) values for CRX and IgG immunoprecipitation (IP) were normalized to input and mock subtracted. The fold enrichment of CRX:IgG was calculated based on the formula shown below. Significant enrichment was determined by testing overall promoter*genotype interactions by two-way ANOVA for repeated measures using SAS 9.3 (SAS Institutes, Cary, NC) (p<0.05, n = 3), as above.

ΔCt = (Ct[CRX or IgG]-Ct[Input])

ΔΔCt = ΔCt[CRX or IgG]- ΔCt[mock]

Fold enrichment = ((2^−ΔΔCt CRX^)/(2^−ΔΔCt IgG^)

## Supporting Information

Figure S1 Genotyping of *E168d2* and *R90W* mice by PCR. Mice were genotyped for the presence of *Crx WT*, *E168d2* or *R90W* alleles by PCR amplification of genomic mouse *Crx* using allele specific primers (Table S1, Panel C). **A**. Specific amplification of *Crx WT* and *E168d2* alleles is shown here in *WT*, *E168d2/+*, *E168d2/d2* and −/− mice. For the *E168d2* colony: the primers E168 Mut-F and E168 WT-F, which are specific to the *E168d2* and *WT* alleles, respectively, were paired with a common reverse primer E168-R for PCR amplification. **B**. Specific amplification of *R90W* and *Crx WT* alleles is shown here in *WT*, *R90W/+*, *R90W/W* and −/− mice. For the *R90W* colony: the primers R90 mut-F and R90 WT-F, which are specific to the *R90W* and *WT* alleles, respectively, were paired with a common reverse primer R90-R for PCR amplification. In addition, non-allele specific total *Crx* was amplified using the primers Total Crx F/R and the presence or absence of *neo* was detected using the primers neo F/R (Data not shown). No amplification of *Crx* is detectable in −/− mice, but *Neo* is present. DNA ladder: 100 bp (New England Biolabs). **C**. Schematic diagram of Exon 4 of *Crx* shows the location of the primer sets for *Crx WT*, *E168d2* and *R90W* genotyping. Numbers refer to nucleotide positions relative to the transcription start site.
(TIF)

Figure S2 Detection of programmed cell death in *E168d2*, *R90W* and *−/−* mice. **A–O**. Cells undergoing programmed cell death were detected by fluorescent TUNEL staining of paraffin embedded sagittal sections. *WT*, *E168d2/d2*, *R90W/W* and *−/−* retinas were assessed at P21 (**A–D**) and P35 (**F–I**). TUNEL+ cells (white arrows) in the ONL of *WT*, *E168d2/d2*, *R90W/W* and −/− retinas were quantified at P21 (**E**) and P35 (**J**). **K–N**. *WT*, *E168d2/+*, *E168d2neo/+*, and *R90W/+* retinas were assessed at P35. **O**. Quantification of TUNEL+ cells in P35 *WT*, *E168d2/+*, *E168d2neo/+*, and *R90W/+* retinas confirms increased programmed cell death in *E168d2/+* retinas (*p<0.05; Error bars: STDEV).
(TIF)

Figure S3 Retinal function is ablated in homozygous *E168d2/d2* and *R90W/W* mice. **A–C**. Retinal function in 1 mo *WT*, *E168d2/d2* and *R90W/W* mice was measured by electroretinogram. Average peak amplitudes of dark-adapted A-waves and B-waves and light-adapted B-waves are shown. (*p<0.05; brackets indicate all enclosed data points are significantly different from WT; Error bars: SEM).
(TIF)

Figure S4 *E168d2/+* mice have disorganized nuclear architecture. **A–F**. Transmission electron micrographs of P21 WT, *E168d2/+* and *E168d2neo/+* retinas showing the ONL nuclei in the regions proximal to inner segments (IS) (**A–C**) or OPL (**D–F**). White arrows indicate cone nuclei in *WT* (**A**) and cone-like nuclei in mutant retinas (**B, C, E**). *E168d2/+* have many ONL nuclei with decondensed chromatin (**B, E** white arrows) which are either displaced cones or rods with disorganized nuclear architecture. *E168d2neo/+* also have several photoreceptors with ‘cone-like’ patterns that are mislocalized to the middle and inner ONL (**C,** white arrows). Highly electron dense nuclei (white pentagon), corresponding with pyknotic nuclei, were identified in the ONL of *E168d2/+* (**E**) and *E168d2neo/+* (**F**) samples but not in *WT* (**D**). **G**. Quantification of condensed heterochromatin as a percentage of the total nuclear area shows a significant reduction of rod heterochromatin in *E168d2/+* but not *E168d2neo/+* retinas (*p<0.05; Error bars: SEM). Image scale bars: 2 µM.
(TIF)

Figure S5 Abnormal localization of cone nuclei in *E168d2/+* and *E168d2neo/+* retinas. The distribution of cone nuclei in the ONL of P14 and 1 mo *WT*, *E168d2/+* and *E168d2neo/+* retinas was assessed by staining paraffin embedded sagittal sections with CARR and DAPI (Figure 5 A–D). The number of nuclei counted in each ONL zone (OONL, MONL and IONL) at P14 (**A**) and 1 mo (**B**) are graphed here. (*p<0.05; Error bars: STDEV).
(TIF)

Table S1 PCR Primer Pairs for Genotyping, qRT-PCR and qChIP. List of oligonucleotides used for all PCR-based analyses.
(XLSX)

Table S2 Expression of genes downregulated in P10 homozygous *Crx E168d2neo*, *R90Wneo* and/or *−/−*. Differential expression analyses of downregulated genes from P10 expression microarrays.
(XLSX)

Table S3 Expression of genes upregulated in P10 homozygous *Crx E168d2neo*, *R90Wneo* and/or *−/−*. Differential expression analyses of upregulated genes from P10 expression microarrays.
(XLSX)

Table S4 Downregulated RP, CoRD, LCA disease genes in P10 homozygous *Crx E168d2neo* and *R90Wneo* mice. List of human genes associated with RP, CoRD or LCA whose mouse homologs were identified as being significantly downregulated in P10 microarray of P10 *E168d2neo/neo* or *R90Wneo/Wneo* mice.
(XLSX)

Table S5 qRT-PCR Expression in P10 mouse retinas. ∧Overall p-value for each primer set was tested in heterozygous and homozygous mice; p<0.09 was considered marginally significant. For all comparisons to *WT*: *p<0.05; reference genes *Tuba1b* and *Ubb*.
(XLSX)

Table S6 qRT-PCR Expression in P21 mouse retinas. ∧Overall p-value for each primer set was tested in heterozygous; p<0.09 was considered marginally significant. For all comparisons to *WT*: *p<0.05; reference genes *Tuba1b* and *Ubb*.
(XLSX)

Table S7 ERG test parameters. Specific testing parameters used for ERG assays.
(XLSX)

## References

[1] Chen S, Wang QL, Nie Z, Sun H, Lennon G, et al (1997) Crx, a novel Otx-like paired-homeodomain protein, binds to and transactivates photoreceptor cell-specific genes. Neuron 19(5): 1017–30. 9390516 10.1016/s0896-6273(00)80394-3

[2] Furukawa T, Morrow EM, Cepko CL (1997) Crx, a novel otx-like homeobox gene, shows photoreceptor-specific expression and regulates photoreceptor differentiation. Cell 91: 531–541. 9390562 10.1016/s0092-8674(00)80439-0

[3] Furukawa T, Morrow EM, Li T, Davis FC, Cepko CL (1999) Retinopathy and attenuated circadian entrainment in *Crx*-deficient mice. Nat Genet 23 4: 466–470. 10581037 10.1038/70591

[4] Chau KY, Chen S, Zack DJ, Ono SJ (2000) Functional domains of the cone-rod homeobox (CRX) transcription factor. The Journal of biological chemistry 275 47: 37264–70. 10984472 10.1074/jbc.M002763200

[5] Kimura A, Singh D, Wawrousek E, Kikuchi M, Nakamura M, et al (2000) Both PCE-1/RX and OTX/CRX Interactions Are Necessary for Photoreceptor-specific Gene Expression. J Biol Chem 275: 1152–1160. 10625658 10.1074/jbc.275.2.1152

[6] Langmann T, Lai CCL, Weigelt K, Tam BM, Warneke-Wittstock R, et al (2008) CRX controls retinal expression of the X-linked juvenile retinoschisis (RS1) gene. Nucleic acids research 36 20: 6523–34. 18927113 10.1093/nar/gkn737 PMC2582616

[7] Peng GH, Chen S (2007) Crx activates opsin transcription by recruiting HAT-containing co-activators and promoting histone acetylation. Hum Mol Genet 16 20: 2433–2452. 17656371 10.1093/hmg/ddm200 PMC2276662

[8] Hennig AK, Peng GH, Chen S (2008) Regulation of photoreceptor gene expression by Crx-associated transcription factor network. Brain Res 1192: 114–133. 17662965 10.1016/j.brainres.2007.06.036 PMC2266892

[9] Peng GH, Chen S (2011) Active opsin loci adopt intrachromosomal loops that depend on the photoreceptor transcription factor network. Proc Natl Acad Sci USA 108 43: 17821–6. 22006320 10.1073/pnas.1109209108 PMC3203788

[10] Fei Y, Hughes TE (2000) Nuclear trafficking of photoreceptor protein crx: the targeting sequence and pathologic implications. Investigative ophthalmology & visual science 41 10: 2849–56. 10967037

[11] Mitton KP, Swain PK, Chen S, Xu S, Zack DJ, et al (2000) The leucine zipper of NRL interacts with the CRX homeodomain. A possible mechanism of transcriptional synergy in rhodopsin regulation. J Biol Chem 275 38: 29794–9. 10887186 10.1074/jbc.M003658200

[12] Nichols LL, Alur RP, Boobalan E, Sergeev YV, Caruso RC, et al (2010) Two novel CRX mutant proteins causing autosomal dominant Leber congenital amaurosis interact differently with NRL. Human mutation 31 6: E1472–83. 20513135 10.1002/humu.21268 PMC2952391

[13] Peng GH, Ahmad O, Ahmad F, Liu J, Chen S (2005) The photoreceptor-specific nuclear receptor Nr2e3 interacts with Crx and exerts opposing effects on the transcription of rod versus cone genes. Hum Mol Genet 14: 747–764. 15689355 10.1093/hmg/ddi070

[14] Roduit R, Escher P, Schorderet DF (2009) Mutations in the DNA-binding domain of NR2E3 affect in vivo dimerization and interaction with CRX. PLoS ONE 4 10: e7379. 19823680 10.1371/journal.pone.0007379 PMC2757917

[15] Sanyal S, Jansen HG (1981) Absence of receptor outer segments in the retina of rds mutant mice. Neurosci Lett 21: 23–26. 7207866 10.1016/0304-3940(81)90051-3

[16] Humphries MH, Rancourt D, Farrar GJ, Kenna P, Hazel M, et al (1997) Retinopathy induced in mice by targeted disruption of the rhodopsin gene. Nature Genet 15: 216–219. 9020854 10.1038/ng0297-216

[17] Morrow EM, Furukawa T, Raviola E, Cepko CL (2005) Synaptogenesis and outer segment formation are perturbed in the neural retina of Crx mutant mice. BMC Neurosci 6: 5. 15676071 10.1186/1471-2202-6-5 PMC548520

[18] Livesey FJ, Furukawa T, Steffen MA, Church GM, Cepko CL (2000) Microarray analysis of the transcriptional network controlled by the photoreceptor homeobox gene Crx. Curr Biol 10 6: 301–310. 10744971 10.1016/s0960-9822(00)00379-1

[19] Hsiau TH, Diaconu C, Myers CA, Lee J, Cepko CL, et al (2007) The cis-regulatory logic of the mammalian photoreceptor transcriptional network. PLoS ONE 2 7: 2e643. 10.1371/journal.pone.0000643 PMC1916400 17653270

[20] Blackshaw S, Fraioli RE, Furukawa T, Cepko CL (2001) Comprehensive analysis of photoreceptor gene expression and the identification of candidate retinal disease genes. Cell 107 5: 579–589. 11733058 10.1016/s0092-8674(01)00574-8

[21] Corbo JC, Lawrence KA, Karlstetter M, Myers CA, Abdelaziz M, et al (2010) CRX ChIP-seq reveals the cis-regulatory architecture of mouse photoreceptors. Genome Research 20 11: 1512–1525. 20693478 10.1101/gr.109405.110 PMC2963815

[22] Rivolta C, Berson EL, Dryja TP (2001) Dominant Leber congenital amaurosis, cone-rod degeneration, and retinitis pigmentosa caused by mutant versions of the transcription factor CRX. Human mutation 18 6: 488–98. 11748842 10.1002/humu.1226

[23] Sohocki MM, Sullivan LS, Mintz-Hittner HA, Birch D, Heckenlively JR, et al (1998) A range of clinical phenotypes associated with mutations in CRX, a photoreceptor transcription-factor gene. Am J Hum Genet 63 5: 1307–1315. 9792858 10.1086/302101 PMC1377541

[24] Jacobson SG, Cideciyan AV, Huang Y, Hanna DB, Freund CL, et al (1998) Retinal degenerations with truncation mutations in the cone-rod homeobox (CRX) gene. Invest Ophthalmol Vis Sci 39 12: 2417–2426. 9804150

[25] Hanein S, Perrault I, Gerber S, Tanguy G, Barbet F, et al (2004) Leber congenital amaurosis: comprehensive survey of the genetic heterogeneity, refinement of the clinical definition, and genotype-phenotype correlations as a strategy for molecular diagnosis. Human mutation 23: 306–317. 15024725 10.1002/humu.20010

[26] Dharmaraj SR, Silva ER, Pina a L, Li YY, Yang JM, et al (2000) Mutational analysis and clinical correlation in Leber congenital amaurosis. Ophthalmic genetics 21: 135–150. 11035546

[27] Galvin JA, Fishman GA, Stone EM, Koenekoop RK (2005) Evaluation of genotype-phenotype associations in Leber Congenital Amaurosis. Retina 25 7: 919–29. 16205573 10.1097/00006982-200510000-00016

[28] Perrault I, Hanein S, Gerber S, Barbet F, Dufier JL, et al (2003) Evidence of autosomal dominant Leber congenital amaurosis (LCA) underlain by a CRX heterozygous null allele. J Med Genet 40 7: e90. 12843339 10.1136/jmg.40.7.e90 PMC1735514

[29] Nakamura M, Ito S, Miyake Y (1998) Novel De Novo Mutation in CRX Gene in a Japanese Patient with Leber Congenital Amaurosis. American Journal of Ophthalmology 24: 465–467. 10.1016/s0002-9394(02)01542-8 12208271

[30] Zhang Q, Li S, Guo X, Guo L, Xiao X, et al (2001) Screening for CRX gene mutations in Chinese patients with Leber congenital amaurosis and mutational phenotype. Ophthalmic Genet 22: 89–96. 11449318 10.1076/opge.22.2.89.2227

[31] Lotery AJ, Namperumalsamy P, Jacobson SG, Weleber RG, Fishman GA, et al (2000) Mutation analysis of 3 genes in patients with Leber congenital amaurosis. Archives of ophthalmology 118 4: 538–43. 10766140 10.1001/archopht.118.4.538

[32] Wang P, Guo X, Zhang Q (2007) Further evidence of autosomal-dominant Leber congenital amaurosis caused by heterozygous CRX mutation. Graefe's archive for clinical and experimental ophthalmology 245: 1401–1402. 10.1007/s00417-007-0554-0 17347810

[33] Huang L, Xiao X, Li S, Jia X, Wang P, et al (2012) *CRX* variants in cone-rod dystrophy and mutation overview. Biochemical and biophysical research communications 426 4: 498–503. 22960069 10.1016/j.bbrc.2012.08.110

[34] Tzekov RT, Liu Y, Sohocki MM, Zack DJ, Daiger SP, et al (2001) Autosomal dominant retinal degeneration and bone loss in patients with a 12-bp deletion in the CRX gene. Investigative ophthalmology & visual science 42: 1319–1327. 11328746 PMC2581450

[35] Koenekoop RK, Loyer M, Dembinska O, Beneish R (2002) Mutation report Visual improvement in Leber congenital amaurosis and the CRX genotype. Ophthalmic Genet 23: 49–60. 11910559 10.1076/opge.23.1.49.2200

[36] Paunescu K, Preising MN, Janke B, Wissinger B, Lorenz B (2007) Genotype-phenotype correlation in a German family with a novel complex CRX mutation extending the open reading frame. Ophthalmology 114: 1348–1357.e1. 17320181 10.1016/j.ophtha.2006.10.034

[37] Tzekov RT, Sohocki MM, Daiger SP, Birch DG (2000) Visual phenotype in patients with Arg41Gln and ala196+1 bp mutations in the CRX gene. Ophthalmic genetics 21 2: 89–99. 10916183

[38] Walia S, Fishman GA, Jacobson SG, Aleman TS, Koenekoop RK, et al (2010) Visual acuity in patients with Leber's congenital amaurosis and early childhood-onset retinitis pigmentosa. Ophthalmology 117: 1190–1198. 20079931 10.1016/j.ophtha.2009.09.056

[39] den Hollander AI, Roepman R, Koenekoop RK, Cremers FPM (2008) Leber congenital amaurosis: genes, proteins and disease mechanisms. Progress in retinal and eye research 27: 391–419. 18632300 10.1016/j.preteyeres.2008.05.003

[40] Freund CL, Gregory-Evans CY, Furukawa T, Papaioannou M, Looser J, et al (1997) Cone-rod dystrophy due to mutations in a novel photoreceptor-specific homeobox gene (CRX) essential for maintenance of the photoreceptor. Cell 91 4: 543–553. 9390563 10.1016/s0092-8674(00)80440-7

[41] Freund C, Wang QL, Chen S, Muskat B, Wiles C, et al (1998) De novo mutations in the CRX homeobox gene associated with Leber congenital amaurosis. Nature genetics 18 4: 311–312. 9537410 10.1038/ng0498-311

[42] Swain PK, Chen S, Wang QL, Affatigato LM, Coats CL, et al (1997) Mutations in the cone-rod homeobox gene are associated with the cone-rod dystrophy photoreceptor degeneration. Neuron 19 6: 1329–36. 9427255 10.1016/s0896-6273(00)80423-7

[43] Berger W, Kloeckener-Gruissem B, Neidhardt J (2010) The molecular basis of human retinal and vitreoretinal diseases. Progress in retinal and eye research 29 5: 335–75. 20362068 10.1016/j.preteyeres.2010.03.004

[44] Silva E, Yang JM, Li Y, Darmaraj S, Oh S, et al (2000) A *CRX* null mutation is associated with both Leber congenital amaurosis and a normal ocular phenotype. Invest Ophthalmol Vis Sci 41: 2076–2079. 10892846

[45] Swaroop A, Wang QL, Wu W, Cook J, Coats C, et al (1999) Leber congenital amaurosis caused by a homozygous mutation (*R90W*) in the homeodomain of the retinal transcription factor CRX: direct evidence for the involvement of CRX in the development of photoreceptor function. Hum Mol Genet 8 2: 299–305. 9931337 10.1093/hmg/8.2.299

[46] Terrell D, Xie B, Workman M, Mahato S, Zelhof A, et al (2012) OTX2 and CRX rescue overlapping and photoreceptor-specific functions in the Drosophila eye. Developmental dynamics: an official publication of the American Association of Anatomists 241: 215–228. 22113834 10.1002/dvdy.22782 PMC3444242

[47] Chen S, Wang QL, Xu S, Liu I, Li L, et al (2002) Functional analysis of cone-rod homeobox (CRX) mutations associated with retinal dystrophy. Hum Mol Genet 11 8: 873–884. 11971869 10.1093/hmg/11.8.873

[48] Hayashi S, Lewis P, Pevny L, McMahon AP (2002) Efficient gene modulation in mouse epiblast using a Sox2Cre transgenic mouse strain. Mech Dev Suppl 1: S97–S101. 14516668 10.1016/s0925-4773(03)00099-6

[49] Nishida A, Furukawa A, Koike C, Tano Y, Aizawa S, et al (2003) Homeobox gene controls retinal photoreceptor cell fate and pineal gland development. Nature Neuroscience 6 12: 1255–1263. 14625556 10.1038/nn1155

[50] Peachey NS, Ball SL (2003) Electrophysiological analysis of visual function in mutant mice. Documenta ophthalmologica. Advances in ophthalmology 107 1: 13–36. 12906119 10.1023/a:1024448314608

[51] Carter-Dawson LD, LaVail MM (1979) Rods and Cones in the Mouse Retina. Journal of Comparative Neurology 188 2: 245–262. 500858 10.1002/cne.901880204

[52] Chiquet C, Dkhissi-Benyahya O, Chounlamountri N, Szel A, Degrip WJ, et al (2002) Characterization of calbindin-positive cones in primates. Neuroscience 115 4: 1323–33. 12453500 10.1016/s0306-4522(02)00327-5

[53] Rich KA, Zhan Y, Blanks JC (1997) Migration and synaptogenesis of cone photoreceptors in the developing mouse retina. The Journal of comparative neurology 388 1: 47–63. 9364238

[54] Mieziewska KE, Van Veen T, Murray JM, Aguirre GD (1991) Rod and cone specific domains in the interphotoreceptor matrix. The Journal of Comparative Neurology 308 3: 371–80. 1865006 10.1002/cne.903080305

[55] Szél A, Röhlich P, Caffé AR, Van Veen T (1996) Distribution of cone photoreceptors in the mammalian retina. Microscopy research and technique 35 6: 445–62. 9016448 10.1002/(SICI)1097-0029(19961215)35:6<445::AID-JEMT4>3.0.CO;2-H

[56] Applebury ML, Antoch MP, Baxter LC, Chun LL, Falk JD, et al (2000) The murine cone photoreceptor: a single cone type expresses both S and M opsins with retinal spatial patterning. Neuron 27: 513–523. 11055434 10.1016/s0896-6273(00)00062-3

[57] Furukawa A, Koike C, Lippincott P, Cepko CL, Furukawa T (2002) The mouse Crx 5′-upstream transgene sequence directs cell-specific and developmentally regulated expression in retinal photoreceptor cells. The Journal of Neuroscience 22 5: 1640–7. 11880494 10.1523/JNEUROSCI.22-05-01640.2002 PMC6758905

[58] Roberts MR, Hendrickson A, McGuire CR, Reh TA (2005) Retinoid X receptor (gamma) is necessary to establish the S-opsin gradient in cone photoreceptors of the developing mouse retina. Investigative ophthalmology & visual science 46 8: 2897–904. 16043864 10.1167/iovs.05-0093

[59] Roberts MR, Srinivas M, Forrest D, Morreale de Escobar G, Reh TA (2006) Making the gradient: thyroid hormone regulates cone opsin expression in the developing mouse retina. Proc Natl Acad Sci USA 103 16: 6218–23. 16606843 10.1073/pnas.0509981103 PMC1458858

[60] Menotti-Raymond M, Deckman KH, David VA, Myrkalo J, O'Brien SJ, et al (2010) Mutation discovered in a feline model of human congenital retinal blinding disease. Invest Ophthalmol Vis Sci 51 6: 2852–9. 20053974 10.1167/iovs.09-4261 PMC2891453

[61] Curtis R, Barnett K, Leon A (1987) An Early-Onset Retinal Dystrophy With Dominant Inheritance in the Abyssinian Cat. IOVS 28: 131–139. 3804643

[62] Omori Y, Katoh K, Sato S, Muranishi Y, Chaya T, et al (2011) Analysis of transcriptional regulatory pathways of photoreceptor genes by expression profiling of the Otx2-deficient retina. PloS one 6 5: e19685. 21602925 10.1371/journal.pone.0019685 PMC3094341

[63] Koike C, Nishida A, Ueno S, Saito H, Sanuki R, et al (2007) Functional roles of Otx2 transcription factor in postnatal mouse retinal development. Mol Cell Biol 27 23: 8318–8329. 17908793 10.1128/MCB.01209-07 PMC2169187

[64] Lamba DA, Gust J, Reh TA (2009) Transplantation of human embryonic stem cell-derived photoreceptors restores some visual function in *Crx*-deficient mice. Cell stem cell 4 1: 73–9. 19128794 10.1016/j.stem.2008.10.015 PMC2713676

[65] Chadderton N, Millington-Ward S, Palfi A, O'Reilly M, Tuohy G, et al (2009) Improved retinal function in a mouse model of dominant retinitis pigmentosa following AAV-delivered gene therapy. Molecular therapy: the journal of the American Society of Gene Therapy 17 4: 593–9. 19174761 10.1038/mt.2008.301 PMC2835099

[66] O'Reilly M, Palfi A, Chadderton N, Millington-Ward S, Ader M, et al (2007) RNA interference-mediated suppression and replacement of human rhodopsin in vivo. American journal of human genetics 81 1: 127–35. 17564969 10.1086/519025 PMC1950918

[67] Wang X, Xu S, Rivolta C, Li LY, Peng GH, et al (2002) Barrier to autointegration factor interacts with the cone-rod homeobox and represses its transactivation function. The Journal of Biological Chemistry 277 45: 43288–300. 12215455 10.1074/jbc.M207952200

[68] Chen S, Peng GH, Wang X, Smith AC, Grote SK, et al (2004) Interference of Crx-dependent transcription by ataxin-7 involves interaction between the glutamine regions and requires the ataxin-7 carboxy-terminal region for nuclear localization. Hum Molec Genet 13: 53–67. 14613968 10.1093/hmg/ddh005

