# Supplementary figures and images for: Mechanistically Distinct Mouse Models for CRX-Associated Retinopathy
Source: PLoS Genet. 2014 Feb 6;10(2):e1004111. doi: 10.1371/journal.pgen.1004111 (PMC3916252; doi:10.1371/journal.pgen.1004111)

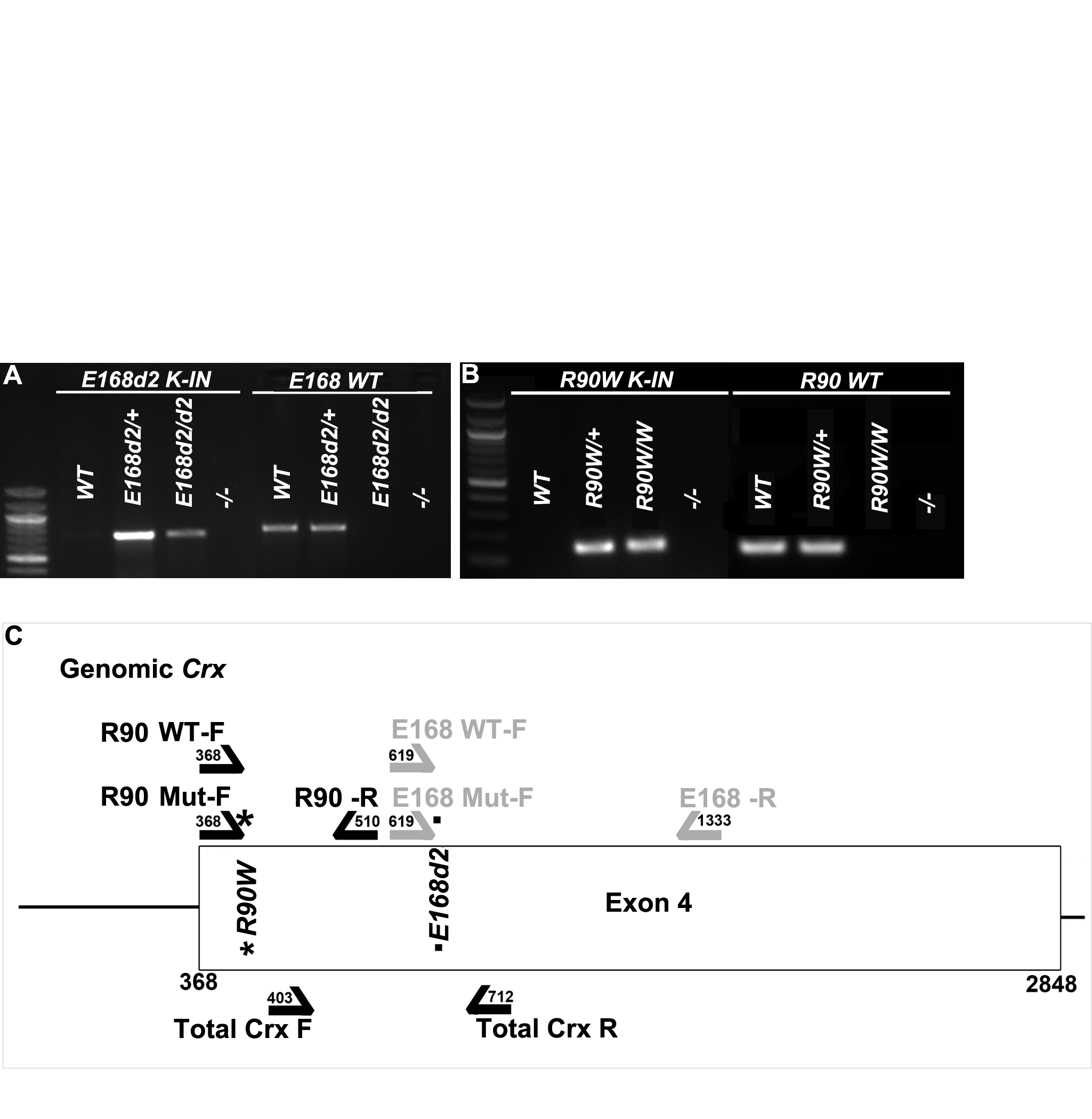

Supplement: Figure S1 — Genotyping of E168d2 and R90W mice by PCR. Mice were genotyped for the presence of Crx WT, E168d2 or R90W alleles by PCR amplification of genomic mouse Crx using allele specific primers (Table S1, Panel C). A. Specific amplification of Crx WT and E168d2 alleles is shown here in WT, E168d2/+, E168d2/d2 and −/− mice. For the E168d2 colony: the primers E168 Mut-F and E168 WT-F, which are specific to the E168d2 and WT alleles, respectively, were paired with a common reverse primer E168-R for PCR amplification. B. Specific amplification of R90W and Crx WT alleles is shown here in WT, R90W/+, R90W/W and −/− mice. For the R90W colony: the primers R90 mut-F and R90 WT-F, which are specific to the R90W and WT alleles, respectively, were paired with a common reverse primer R90-R for PCR amplification. In addition, non-allele specific total Crx was amplified using the primers Total Crx F/R and the presence or absence of neo was detected using the primers neo F/R (Data not shown). No amplification of Crx is detectable in −/− mice, but Neo is present. DNA ladder: 100 bp (New England Biolabs). C. Schematic diagram of Exon 4 of Crx shows the location of the primer sets for Crx WT, E168d2 and R90W genotyping. Numbers refer to nucleotide positions relative to the transcription start site. (TIF) [file pgen.1004111.s001.tif]

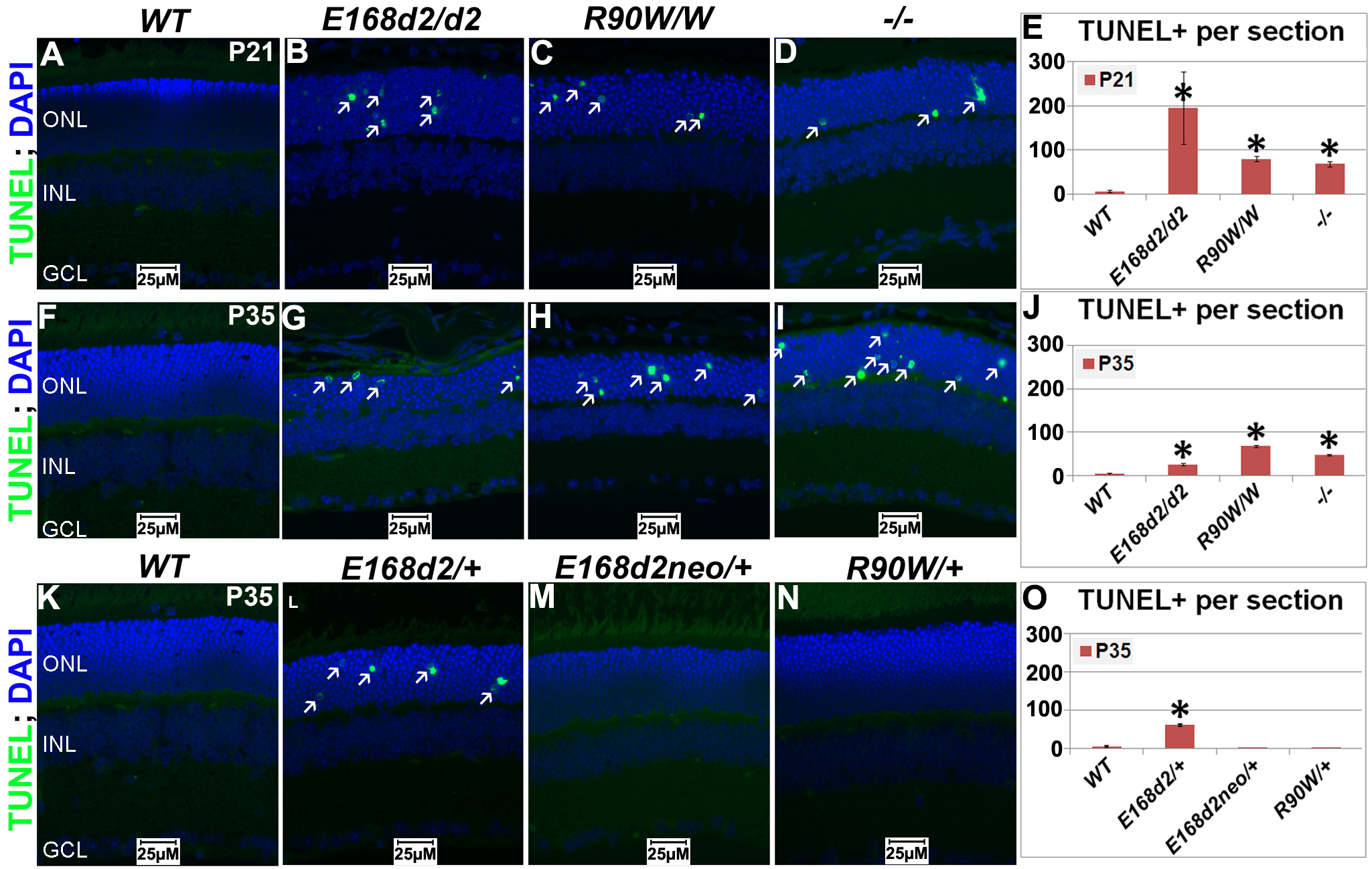

Supplement: Figure S2 — Detection of programmed cell death in E168d2, R90W and −/− mice. A–O. Cells undergoing programmed cell death were detected by fluorescent TUNEL staining of paraffin embedded sagittal sections. WT, E168d2/d2, R90W/W and −/− retinas were assessed at P21 (A–D) and P35 (F–I). TUNEL+ cells (white arrows) in the ONL of WT, E168d2/d2, R90W/W and −/− retinas were quantified at P21 (E) and P35 (J). K–N. WT, E168d2/+, E168d2neo/+, and R90W/+ retinas were assessed at P35. O. Quantification of TUNEL+ cells in P35 WT, E168d2/+, E168d2neo/+, and R90W/+ retinas confirms increased programmed cell death in E168d2/+ retinas (*p<0.05; Error bars: STDEV). (TIF) [file pgen.1004111.s002.tif]

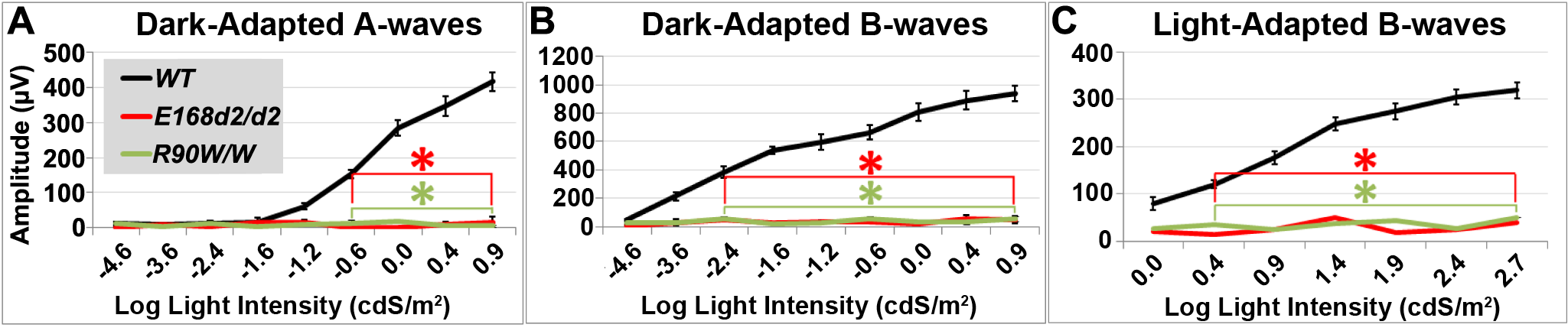

Supplement: Figure S3 — Retinal function is ablated in homozygous E168d2/d2 and R90W/W mice. A–C. Retinal function in 1 mo WT, E168d2/d2 and R90W/W mice was measured by electroretinogram. Average peak amplitudes of dark-adapted A-waves and B-waves and light-adapted B-waves are shown. (*p<0.05; brackets indicate all enclosed data points are significantly different from WT; Error bars: SEM). (TIF) [file pgen.1004111.s003.tif]

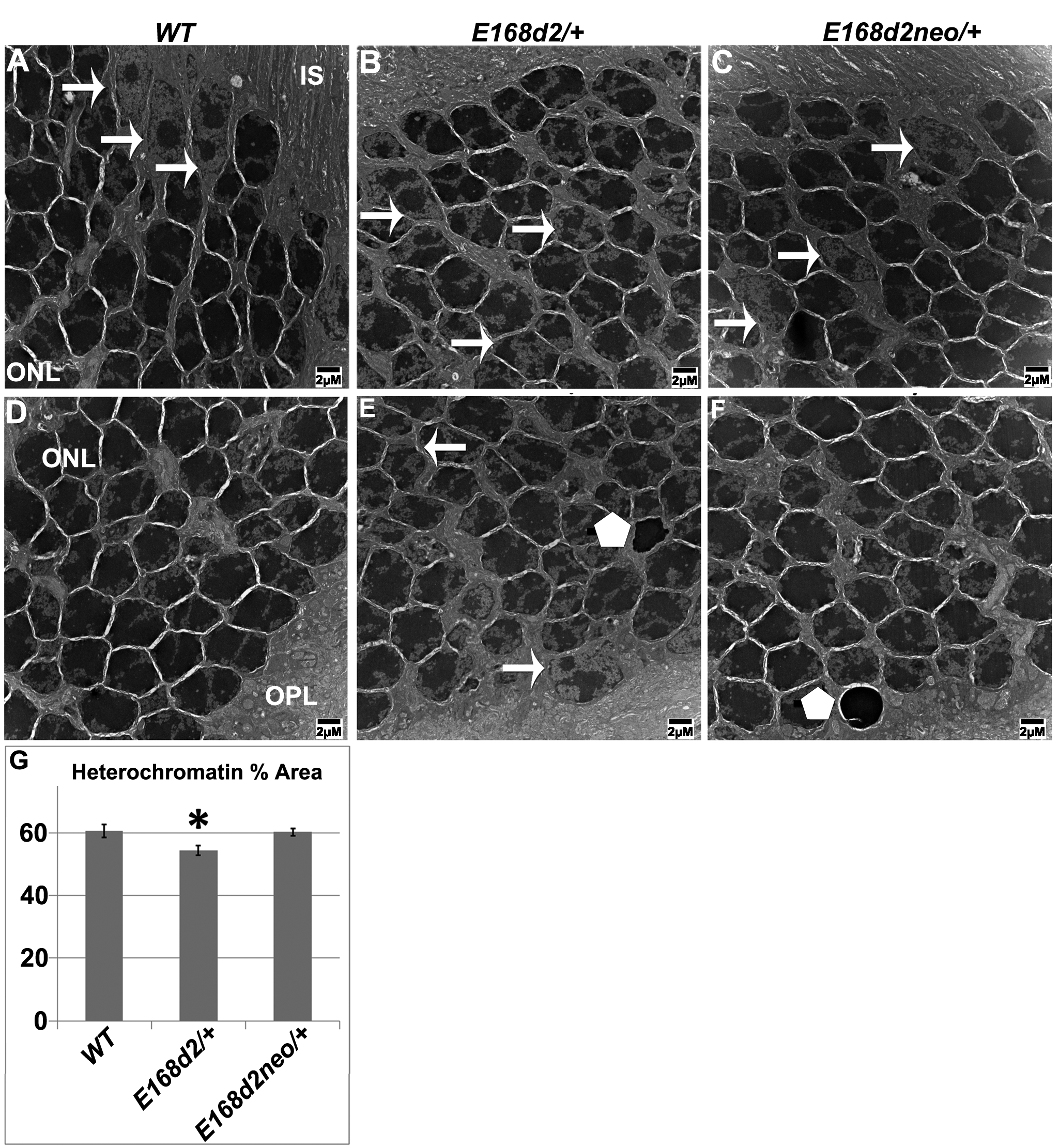

Supplement: Figure S4 — E168d2/+ mice have disorganized nuclear architecture. A–F. Transmission electron micrographs of P21 WT, E168d2/+ and E168d2neo/+ retinas showing the ONL nuclei in the regions proximal to inner segments (IS) (A–C) or OPL (D–F). White arrows indicate cone nuclei in WT (A) and cone-like nuclei in mutant retinas (B, C, E). E168d2/+ have many ONL nuclei with decondensed chromatin (B, E white arrows) which are either displaced cones or rods with disorganized nuclear architecture. E168d2neo/+ also have several photoreceptors with ‘cone-like’ patterns that are mislocalized to the middle and inner ONL (C, white arrows). Highly electron dense nuclei (white pentagon), corresponding with pyknotic nuclei, were identified in the ONL of E168d2/+ (E) and E168d2neo/+ (F) samples but not in WT (D). G. Quantification of condensed heterochromatin as a percentage of the total nuclear area shows a significant reduction of rod heterochromatin in E168d2/+ but not E168d2neo/+ retinas (*p<0.05; Error bars: SEM). Image scale bars: 2 µM. (TIF) [file pgen.1004111.s004.tif]

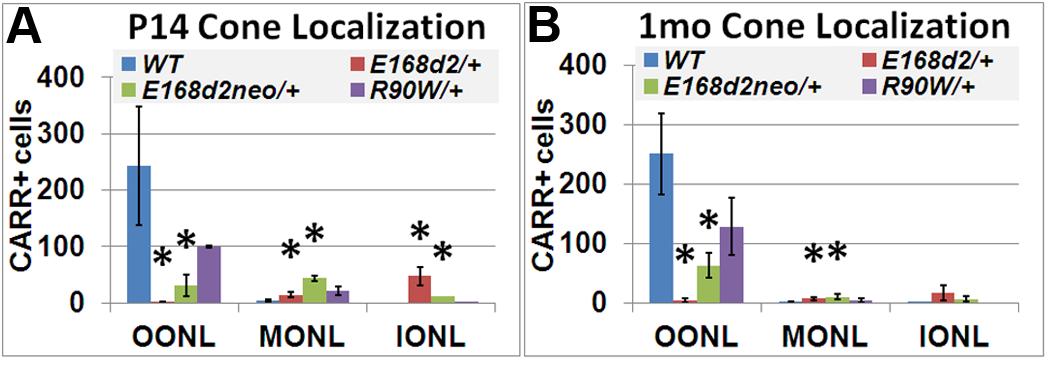

Supplement: Figure S5 — Abnormal localization of cone nuclei in E168d2/+ and E168d2neo/+ retinas. The distribution of cone nuclei in the ONL of P14 and 1 mo WT, E168d2/+ and E168d2neo/+ retinas was assessed by staining paraffin embedded sagittal sections with CARR and DAPI (Figure 5 A–D). The number of nuclei counted in each ONL zone (OONL, MONL and IONL) at P14 (A) and 1 mo (B) are graphed here. (*p<0.05; Error bars: STDEV). (TIF) [file pgen.1004111.s005.tif]
